# Supplementary material for: The unique Legionella longbeachae capsule favors intracellular replication and immune evasion
Source: PLoS Pathog. 2024 Sep 11;20(9):e1012534. doi: 10.1371/journal.ppat.1012534 (PMC11419355; doi:10.1371/journal.ppat.1012534)
Supplement: S1 Table — (PDF) [file ppat.1012534.s001.pdf]

Supplementary Table 1 List of the highest hits of the *L. longbeachae* capsule cluster genes against the NCBI database (Trembl).

Genomic Object Editor: llo3148

PB id	Ident %	Eval	Gene	Description	EC number	Keywords	Organism
Q48462	56.81	0	manC	Mannose-1-phosphate guanylyltransferase	2.7.7.13	Capsule biogenesis/degradation,	Klebsiella pneumoniae
Q01410	57.08	0	manC	Mannose-1-phosphate guanylyltransferase	2.7.7.13	GTP-binding, Lipopolysaccharide	Salmonella montevideo
P37753	56.38	0	manC	Mannose-1-phosphate guanylyltransferase	2.7.7.13	GTP-binding, Lipopolysaccharide	Escherichia coli
P26404	56.13	0	rfbM	Mannose-1-phosphate guanylyltransferase RfbM	2.7.7.13	Direct protein sequencing, GTP-binding,	Salmonella typhimurium (strain LT2 / SGSC1412 / ATCC 700720)
Q8X7P1	56.14	0	manC1	Mannose-1-phosphate guanylyltransferase 1	2.7.7.13	GTP-binding, Nucleotide-binding,	Escherichia coli O157:H7
P07874	55.25	0	algA	Mannose-6-phosphate isomerase / Mannose-1-phosphate	5.3.1.8, 2.7.7.13	Alginate biosynthesis, Cobalt, Direct protein	Pseudomonas aeruginosa (strain ATCC 15692 / DSM 22644 / CIP 104116 /
P24174	55.93	0	manC	Mannose-1-phosphate guanylyltransferase	2.7.7.13	Capsule biogenesis/degradation,	Escherichia coli (strain K12)
P26340	55.93	0	manC	Mannose-1-phosphate guanylyltransferase ManC	2.7.7.13	Capsule biogenesis/degradation,	Salmonella typhimurium (strain LT2 / SGSC1412 / ATCC 700720)
B0RVK6	56.68	0	xanB	Mannose-6-phosphate isomerase / Mannose-1-phosphate guanylyl	5.3.1.8, 2.7.7.13	Exopolysaccharide synthesis, GTP-binding,	Xanthomonas campestris pv. campestris (strain B100)
O85342	56.03	0	manC2	Mannose-1-phosphate guanylyltransferase 2	2.7.7.13	GTP-binding, Lipopolysaccharide	Escherichia coli O157:H7
P0C7J3	56.47	0	xanB	Mannose-6-phosphate isomerase / Mannose-1-phosphate guanylyl	5.3.1.8, 2.7.7.13	Exopolysaccharide synthesis, GTP-binding,	Xanthomonas campestris pv. campestris (strain ATCC 33913 / DSM
Q07024	56.01	0	rfbA	Putative mannose-1-phosphate guanylyltransferase	2.7.7.13	GTP-binding, Lipopolysaccharide	Vibrio cholerae serotype O1 (strain ATCC 39315 / El Tor Inaba N16961)

Genomic Object Editor: llo3149

PB id	Ident %	Eval	Gene	Description	EC number	Keywords	Organism
D3HMB3	100	0	bexD	Capsule polysaccharide export protein bexD	_	Reference proteome, Signal	Legionella longbeachae serogroup 1 (strain NSW150)
A0A1P8FL76	52.28	2E-127	_	Capsular biosynthesis protein	_	Reference proteome, Signal, Transport	Betaproteobacteria bacterium GR16-43
A0A1I3G2S8	54.64	1E-126	_	Polysaccharide export outer membrane protein	_	Signal	Collimonas sp. OK307
A0A1T4QGS8	51.37	5E-123	_	Polysaccharide export outer membrane protein	_	Signal	Geobacter thiogenes
B3E8Y1	52.47	7E-123	_	Polysaccharide export protein	_	Reference proteome, Signal, Transport	Geobacter lovleyi (strain ATCC BAA-1151 / DSM 17278 / SZ)
Q39JA8	51.61	7E-123	_	Polysaccharide export protein	_	Membrane, Signal, Transmembrane, Transmembrane helix	Burkholderia lata (strain ATCC 17760 / DSM 23089 / LMG 22485 / NCIMB 9086 / R18194 / 383)
A0A1I9YRW4	51.76	6E-122	_	Capsular biosynthesis protein	_	Signal, Transport	Paraburkholderia sprentiae WSM5005
A0A2S5SX59	51.32	1E-121	_	Capsular biosynthesis protein	_	Reference proteome, Signal, Transport	Zhizhongheella caldifontis
A0A0J9DW18	51.1	1E-121	_	Capsular biosynthesis protein	_	Signal	Ralstonia sp. MD27
Q7BMG8	50.68	4E-120	wcbC	Capsular biosynthesis protein	_	Signal	Burkholderia pseudomallei
A0A0F7R2Q8	42.63	1E-98	cpx15D	Capsular polysaccharide export protein D	_	Signal	Actinobacillus pleuropneumoniae
Q44132	42.74	5E-98	cpxD	CpxD	_	Signal	Actinobacillus pleuropneumoniae
A0A059WCS1	42.74	2E-97	cpxD	Sugar ABC transporter substrate-binding protein CpxD	_	Signal	Actinobacillus pleuropneumoniae serovar 8 str. 405
A0A059WNF9	41.84	3E-97	cpxD	Sugar ABC transporter substrate-binding protein CpxD	_	Signal	Actinobacillus pleuropneumoniae
Q9RP76	42.63	1E-95	cpxD	CpxD	_	Signal	Mannheimia haemolytica
Q7WS64	40.53	3E-93	bexD	BexD	_	Signal	Haemophilus influenzae
Q714V0	40.79	2E-92	bexD	BexD	_	Signal	Haemophilus influenzae
Q9L9L6	41.42	3E-87	cexD	CexD	_	Signal	Pasteurella multocida
Q8KS22	39.94	1E-86	ctrA	Capsular transport protein	_	Signal	Neisseria meningitidis

Genomic Object Editor: llo3150

PB id	Ident %	Eval	Gene	Description	EC number	Keywords	Organism
D3HMB4	100	1,00E-153	ctrD	Capsule polysaccharide export ATP-binding protein ctrD (Capsular-polysaccharide-transporting ATPase)	3.6.3.38	ATP-binding, Hydrolase, Nucleotide-binding, Reference proteome	Legionella longbeachae serogroup 1 (strain NSW150)
A0A1I9YRU6	67.59	7,00E-108	_	ATP-binding protein	_	ATP-binding, Cell inner membrane, Cell membrane, Membrane, Nucleotide-binding, Translocase	Paraburkholderia sprentiae WSM5005
A0A1V2XQM1	68.52	3,00E-107	_	ATP-binding protein	_	ATP-binding, Cell inner membrane, Cell membrane, Membrane, Nucleotide-binding, Translocase, Transport	Burkholderia cenocepacia
A0A2U9SFH7	68.06	2,00E-106	_	ABC transporter ATP-binding protein	_	ATP-binding, Cell inner membrane, Cell membrane, Membrane, Nucleotide-binding, Translocase, Transport	Burkholderia sp. JP2-270
B2UAD6	68.22	1,00E-105	_	ABC transporter related	_	ATP-binding, Cell inner membrane, Cell membrane, Membrane, Nucleotide-binding, Reference proteome, Translocase	Ralstonia pickettii (strain 12J)
U3GDS8	68.22	1,00E-105	_	ABC transporter domain-containing protein	_	ATP-binding, Cell inner membrane, Cell membrane, Membrane, Nucleotide-binding, Translocase	Ralstonia sp. 5_2_56FAA
A0A117DUD2	68.69	8,00E-105	_	ABC transporter-like protein	_	ATP-binding, Cell inner membrane, Cell membrane, Membrane, Nucleotide-binding, Translocase	Ralstonia sp. NT80
S9RTK8	68.69	8,00E-105	_	ATP-binding protein	_	ATP-binding, Cell inner membrane, Cell membrane, Membrane, Nucleotide-binding, Translocase	Ralstonia sp. AU12-08
A0A1G8E0F9	67.76	4,00E-104	_	Capsular polysaccharide transport system ATP-binding protein	_	ATP-binding, Cell inner membrane, Cell membrane, Membrane, Nucleotide-binding, Translocase	Paraburkholderia phenazinium

Supplementary Table 1

List of the highest hits of the *L. longbeachae* capsule cluster genes against the NCBI database (Trembl).

U2FWZ6	67.45	9,00E-104	_	Capsular polysaccharide ABC transporter, ATP-binding protein KpsT	_	ATP-binding, Cell inner membrane, Cell membrane, Membrane, Nucleotide-binding, Translocase, Transport	Burkholderia sp. AU4i
A0A1N6LM37	67.29	4,00E-103	_	Capsular polysaccharide transport system ATP-binding protein	_	ATP-binding, Cell inner membrane, Cell membrane, Membrane, Nucleotide-binding, Translocase, Transport	Burkholderia sp. GAS332
K7QQ86	63.85	6,00E-97	ctrD	CtrD	_	ATP-binding, Nucleotide-binding	Kingella kingae
B3FHD0	63.85	1,00E-94	ctrD	ATP-binding cassette domain-containing protein	_	ATP-binding, Nucleotide-binding	Neisseria meningitidis
B3FHE0	63.38	9,00E-94	ctrD	ATP-binding protein	3.6.3.38	ATP-binding, Hydrolase, Nucleotide-binding	Neisseria meningitidis
Q9S6K7	61.5	6,00E-92	cpxA	Capsule polysaccharide export transport system ATP-binding protein	_	ATP-binding, Nucleotide-binding	Actinobacillus pleuropneumoniae
Q7B3Y6	60.28	9,00E-91	bexA	BexA	_	ATP-binding, Nucleotide-binding	Haemophilus influenzae
Q44135	61.03	9,00E-91	cpxA	CpxA	_	ATP-binding, Nucleotide-binding	Actinobacillus pleuropneumoniae
O85464	60.09	1,00E-90	hexA	ABC transporter ATP-binding protein	_	ATP-binding, Nucleotide-binding	Pasteurella multocida
Q9L9L9	60.56	3,00E-90	cexA	CexA	_	ATP-binding, Nucleotide-binding	Pasteurella multocida
Q93UJ9	62.25	2,00E-89	wzt2	Wzt2	_	ATP-binding, Cell inner membrane, Cell membrane, Membrane, Nucleotide-binding, Translocase, Transport	Burkholderia pseudomallei
Q9RP79	60.28	2,00E-89	cpxA	Leukotoxin translocation ATP-binding protein LktB	7.4.2.5	ATP-binding, Nucleotide-binding	Mannheimia haemolytica

Genomic Object Editor: llo3151

PB id	Ident %	Eval	Gene	Description	EC number	Keywords	Organism
P32015	45.82	9,00E-74	ctrC	Capsule polysaccharide export inner-membrane protein CtrC	_	Capsule biogenesis/degradation, Cell inner membrane, Polysaccharide transport, Transmembrane	Neisseria meningitidis serogroup B (strain MC58)
P57012	45.82	4,00E-71	ctrC	Capsule polysaccharide export inner-membrane protein CtrC	_	Capsule biogenesis/degradation, Cell inner membrane, Polysaccharide transport, Transmembrane	Neisseria meningitidis serogroup A / serotype 4A (strain DSM 15465 / Z2491)
P19391	40.89	1,00E-66	bexB	Capsule polysaccharide export inner-membrane protein BexB	_	Capsule biogenesis/degradation, Cell inner membrane, Polysaccharide transport, Transmembrane	Haemophilus influenzae
P19390	40.4	8,00E-66	bexB	Capsule polysaccharide export inner-membrane protein BexB	_	Capsule biogenesis/degradation, Cell inner membrane, Polysaccharide transport, Transmembrane	Haemophilus influenzae
P22235	40	6,00E-65	bexB	Capsule polysaccharide export inner-membrane protein BexB	_	Capsule biogenesis/degradation, Cell inner membrane, Polysaccharide transport, Transmembrane	Haemophilus influenzae
P24584	26.64	5,00E-23	kpsM	Polysialic acid transport protein KpsM	_	Cell inner membrane, Cell membrane, Membrane, Transmembrane, Transmembrane helix, Transport	Escherichia coli
P23889	26.23	8,00E-22	kpsM	Polysialic acid transport protein KpsM	_	Cell inner membrane, Cell membrane, Membrane, Transmembrane, Transmembrane helix, Transport	Escherichia coli

Genomic Object Editor: llo3152

PB id	Ident %	Eval	Gene	Description	EC number	Keywords	Organism
P57034	41	5,00E-90	ctrB	Capsule polysaccharide export inner-membrane protein CtrB	_	Capsule biogenesis/degradation, Cell inner membrane, Polysaccharide transport, Transmembrane	Neisseria meningitidis serogroup A / serotype 4A (strain DSM 15465 / Z2491)
P22930	39.72	2,00E-85	bexC	Capsule polysaccharide export inner-membrane protein BexC	_	Capsule biogenesis/degradation, Cell inner membrane, Polysaccharide transport, Transmembrane	Haemophilus influenzae
P32014	38.78	2,00E-83	ctrB	Capsule polysaccharide export inner-membrane protein CtrB	_	Capsule biogenesis/degradation, Cell inner membrane, Polysaccharide transport, Transmembrane	Neisseria meningitidis serogroup B (strain MC58)
P62586	25.07	7,00E-35	kpsE	Capsule polysaccharide export inner-membrane protein KpsE	_	Capsule biogenesis/degradation, Cell inner membrane, Polysaccharide transport, Transmembrane	Escherichia coli
P42501	25.07	2,00E-34	kpsE	Capsule polysaccharide export inner-membrane protein KpsE	_	Capsule biogenesis/degradation, Cell inner membrane, Polysaccharide transport, Transmembrane	Escherichia coli
P43111	23.98	2,00E-18	vexD	Vi polysaccharide export inner-membrane protein VexD	_	Capsule biogenesis/degradation, Cell inner membrane, Polysaccharide transport, Transmembrane	Salmonella typhi

Supplementary Table 1 List of the highest hits of the *L. longbeachae* capsule cluster genes against the NCBI database (Trembl).

Q48452	20.75	0.063	_	Putative tyrosine-protein kinase in cps region	2.7.10.-	ATP-binding, Cell inner membrane, Exopolysaccharide synthesis, Nucleotide-binding, Phosphoprotein, Transferase Transmembrane	Klebsiella pneumoniae
Q9F7B1	21.72	0.25	wzc	Tyrosine-protein kinase wzc	2.7.10.-	ATP-binding, Cell inner membrane, Exopolysaccharide synthesis, Nucleotide-binding, Phosphoprotein, Transferase Transmembrane	Salmonella typhimurium (strain LT2 / SGSC1412 / ATCC 700720)

Genomic Object Editor: llo3153

PB id	Ident %	Eval	Gene	Description	EC number	Keywords	Organism
D3HMB7	100	0	_	Putative glycosyl transferase group 1	_	Reference proteome, Transferase	Legionella longbeachae serogroup 1 (strain NSW150)
F5YHI6	25.36	2,00E-30	_	Glycosyltransferase, family 1	_	Coiled coil, Reference proteome, Transferase	Treponema primitia (strain ATCC BAA-887 / DSM 12427 / ZAS-2)
A0A2P6CAZ8	26.72	3,00E-30	_	Glycos_transf_1 domain-containing protein	_	_	Polaribacter butkevichii
A0A193SEV6	28.15	3,00E-29	_	Putative mannosyltransferase B	_	Glycosyltransferase, Transferase	Klebsiella pneumoniae
I9BN45	27.16	5,00E-29	_	Glycos_transf_1 domain-containing protein	_	Coiled coil	Bacteroides fragilis CL05T12C13
E4VU69	26.98	3,00E-28	_	Glycosyltransferase, group 1 family protein	2.4.-.-	Coiled coil, Glycosyltransferase, Transferase	Bacteroides fragilis 3_1_12
O84909	30.59	0.00000000000007	wbpY	Glycosyltransferase WbpY	_	Transferase	Pseudomonas aeruginosa
Q47594	25.25	0.000000000007	mtfB	Mannosyltransferase	_	Glycosyltransferase, Transferase	Escherichia coli
Q9RMT9	25.25	0.000000000007	wbdB	WbdB	_	_	Klebsiella pneumoniae
C8YZ35	27.32	0.000000009	wejJ	WejJ	_	_	Escherichia coli
M4QPQ6	31.2	0.00000003	wbdA	Mannosyltransferase	_	Glycosyltransferase, Transferase	Escherichia coli
Q9RMU0	34.02	0.0000002	wbdA	WbdA	_	_	Klebsiella pneumoniae
C8YZ32	29.1	0.0000002	wejI	WejI	_	_	Escherichia coli
Q00481	25.59	0.0000002	_	Glycosyltransferase	_	Transferase	Salmonella enterica
Q93CS4	41.54	0.000001	wbaX	Putative glycosyl transferase	_	Transferase	Shigella boydii
Q9LC66	31.53	0.000002	wbdA	Mannosyltransferase	_	Glycosyltransferase, Transferase	Klebsiella pneumoniae
Q47593	31.53	0.000002	mtfA	Mannosyltransferase A	_	Glycosyltransferase, Transferase	Escherichia coli
M4QN28	31.53	0.000002	wbdA	Mannosyltransferase	_	Glycosyltransferase, Transferase	Escherichia coli

Genomic Object Editor: llo3154

PB id	Ident %	Eval	Gene	Description	EC number	Keywords	Organism
D3HMB8	100	0	_	Putative glycosyl transferase, group 1	_	Coiled coil, Reference proteome, Transferase	Legionella longbeachae serogroup 1 (strain NSW150)
A0A254RBS0	36.96	2,00E-76	_	Uncharacterized protein	_	Membrane, Transmembrane, Transmembrane helix	Fibrobacter sp. UWR2
A0A1V4R768	32.1	1,00E-64	_	Glycos_transf_1 domain-containing protein	_	_	Candidatus Cloacimonas sp. 4484_140
A0A2E0ZAU5	34.06	3,00E-56	_	Colanic acid biosynthesis glycosyltransferase WcaL	_	Transferase	Anaerolineaceae bacterium
A0A0P9DL51	31.5	6,00E-52	_	Colanic acid biosynthesis glycosyl transferase	_	Reference proteome, Transferase	Kouleothrix aurantiaca
F2NHK4	32.51	6,00E-51	_	Glycosyl transferase group 1	_	Reference proteome, Transferase	Desulfobacca acetoxidans (strain ATCC 700848 / DSM 11109 / ASRB2)
A0A2V2RIU7	31.47	2,00E-50	_	Colanic acid biosynthesis glycosyltransferase WcaL	_	Transferase	Acidobacteria bacterium
A0A193SBW5	28.34	9,00E-27	wclQ	Glycosyltransferase protein	2.4.1.57	Glycosyltransferase, Transferase	Klebsiella pneumoniae
A4F3K9	26.62	0.0000000000006	aerI	Putative glycosyltransferase	_	Transferase	Planktothrix agardhii NIVA-CYA 126
Q204F0	24.43	0.00000000006	cps2G	Cps1/2G	_	Glycosyltransferase, Transferase	Streptococcus suis
Q9RHD0	25.96	0.00000000007	wbpU	Glycosyl transferase-like protein	2.4.1.21	Glycosyltransferase, Transferase	Pseudomonas aeruginosa
Q6L735	26.35	0.0000000004	_	Glycosyltransferase	_	Glycosyltransferase, Transferase	Streptomyces kanamyceticus
Q8KWP7	29.7	0.000000001	cps9vG	Capsular polysaccharide biosynthesis protein Cps4H	2.4.1.21	Glycosyltransferase, Transferase	Streptococcus pneumoniae
D2KXE7	22.45	0.000000003	epsG	Putative glycosyltransferase	_	Transferase	Lactobacillus fermentum
Q8GJ89	25.38	0.000000004	wbyC	Putative glycosyltransferase	_	Transferase	Yersinia pseudotuberculosis
Q9EVX4	25.2	0.000000007	cpsG	Putative hexose transferase	_	Transferase	Streptococcus salivarius
Q00481	27.03	0.00000001	_	Glycosyltransferase	_	Transferase	Salmonella enterica

Genomic Object Editor: llo3155

PB id	Ident %	Eval	Gene	Description	EC number	Keywords	Organism
D3HMB9	100	0	ugd	UDP-glucose 6-dehydrogenase	1.1.1.22	NAD, Oxidoreductase, Reference proteome	Legionella longbeachae serogroup 1 (strain NSW150)
A0A088U1Y7	65.98	0	_	UDP-glucose 6-dehydrogenase	1.1.1.22	NAD, Oxidoreductase	Burkholderia cenocepacia
B3QQB3	65.46	0	_	UDP-glucose 6-dehydrogenase	1.1.1.22	NAD, Oxidoreductase	Chlorobaculum parvum (strain NCIB 8327)
A0A0B0YZU4	64.95	0	ugd	UDP-glucose 6-dehydrogenase	1.1.1.22	NAD, Oxidoreductase	Escherichia coli
D2ZEC2	65.21	0	_	UDP-glucose 6-dehydrogenase	1.1.1.22	NAD, Oxidoreductase	Enterobacter cancerogenus ATCC 35316
A0A132F643	65.72	0	_	UDP-glucose 6-dehydrogenase	1.1.1.22	NAD, Oxidoreductase	Burkholderia pseudomultivorans
A0A2J0NPG5	64.95	0	_	UDP-glucose 6-dehydrogenase	1.1.1.22	NAD, Oxidoreductase	Enterobacter mori
V3QX60	64.95	0	_	UDP-glucose 6-dehydrogenase	1.1.1.22	NAD, Oxidoreductase	Enterobacter sp. MGH 24
A0A0D7LW76	64.69	0	_	UDP-glucose 6-dehydrogenase	1.1.1.22	NAD, Oxidoreductase	Citrobacter freundii
A0A0M7DFE8	64.69	0	ugd	UDP-glucose 6-dehydrogenase	1.1.1.22	NAD, Oxidoreductase	Enterobacter cloacae
A0A0H3CLX6	64.69	0	_	UDP-glucose 6-dehydrogenase	1.1.1.22	NAD, Oxidoreductase	Enterobacter cloacae subsp. cloacae (strain ATCC 13047 / DSM 30054 / NBRC 13535 / NCDC 279-56)

A0A0J9WZA6	64.18	9,00E-178	ugd	UDP-glucose 6-dehydrogenase	1.1.1.22	3D-structure, NAD, Nucleotide-binding, Oxidoreductase	Klebsiella pneumoniae subsp. pneumoniae NTUH-K2044
O06519	63.92	5,00E-177	ugd	UDP-glucose 6-dehydrogenase	1.1.1.22	NAD, Oxidoreductase	Escherichia coli
Q6U8B9	63.66	5,00E-177	ugd	UDP-glucose 6-dehydrogenase	1.1.1.22	NAD, Oxidoreductase	Raoultella terrigena
Q56625	62.11	6,00E-176	_	UDP-glucose 6-dehydrogenase	1.1.1.22	NAD, Oxidoreductase	Vibrio cholerae O139
Q9RP54	63.14	1,00E-174	ugd	UDP-glucose 6-dehydrogenase	1.1.1.22	NAD, Oxidoreductase	Escherichia coli
M9P0X4	60.31	1,00E-169	ugd	UDP-glucose 6-dehydrogenase	1.1.1.22	NAD, Oxidoreductase	Providencia alcalifaciens

Genomic Object Editor: llo3156

PB id	Ident %	Eval	Gene	Description	EC number	Keywords	Organism
D3HMC0	100	0	_	Putative glycosyl transferase group 1	_	Reference proteome, Transferase	Legionella longbeachae serogroup 1 (strain NSW150)
H2FUE7	47.1	0	_	Glycosyl transferase group 1	_	Reference proteome, Transferase	Oceanimonas sp. (strain GK1)
A4BRU1	46.78	0	_	Glycosyltransferase	_	Reference proteome, Transferase	Nitrococcus mobilis Nb-231
A0A1I3JCB7	36.83	2,00E-114	_	Glycosyl transferases group 1	_	Transferase	Nitrosomonas sp. Nm34
A0A0P9U3K9	36.88	6,00E-113	_	Glycos_transf_1 domain-containing protein	_	_	Pseudomonas syringae pv. helianthi
A0A101D0Z2	35.6	1,00E-110	_	Glycos_transf_1 domain-containing protein	_	_	Halomonas sp. 54_146
A0A1H2QA17	35.97	1,00E-107	_	Glycosyltransferase involved in cell wall bisynthesis	_	Transferase	Nitrosomonas communis
A0A1I4MRS6	36.35	1,00E-106	_	Glycosyl transferases group 1	_	Transferase	Nitrosomonas communis
K9E0G4	35.75	2,00E-105	_	Glycos_transf_1 domain-containing protein	_	Reference proteome	Massilia timonae CCUG 45783
Q9RQU9	29.38	1,00E-31	wbqA	Putative perosamine transferase	_	Transferase	Caulobacter vibrioides
L7S459	34.65	0.00000004	gwEuk	Glycose transferase group 1 domain protein	_	Transferase	Phytophthora hibernalis
M4QPQ6	28.67	0.00000006	wbdA	Mannosyltransferase	_	Glycosyltransferase, Transferase	Escherichia coli
L7S4R0	34.65	0.0000001	gwEuk	Glycose transferase group 1 domain protein	_	Transferase	Phytophthora ramorum
L7SZH1	33.66	0.0000003	gwEuk 30.30.1	Glycosyltransferase group 1 domain	_	Transferase	Phytophthora lateralis
Q9RMU0	23.38	0.000002	wbdA	WbdA	_	_	Klebsiella pneumoniae

Genomic Object Editor: llo3157

PB id	Ident %	Eval	Gene	Description	EC number	Keywords	Organism
D3HMC1	100	0	_	Uncharacterized protein	_	Coiled coil, Reference proteome	Legionella longbeachae serogroup 1 (strain NSW150)
A0A2S4KG72	25.84	1,00E-33	_	Uncharacterized protein	_	Coiled coil	Diaphorobacter sp. LR2014-1
A0A2D4Y0U4	22.8	3,00E-24	_	Uncharacterized protein	_	Coiled coil	Sphingomonadaceae bacterium
A0A011NI14	28.01	1,00E-21	_	Uncharacterized protein	_	Coiled coil	Candidatus Accumulibacter sp. BA-92
A0A2T7UAV0	23.98	2,00E-21	_	Uncharacterized protein	_	Coiled coil	Limnohabitans planktonicus II-D5
A0A1H8X4C5	34.33	1,00E-17	_	Uncharacterized protein	_	_	Pseudomonas sp. Snoq117.2
A0A0K8NZ38	26.47	3,00E-17	_	Uncharacterized protein	_	Coiled coil, Reference proteome	Ideonella sakaiensis (strain NBRC 110686 / TISTR 2288 / 201-F6)
D0QYN0	23.73	0.000000000001	ccbE	CcbE	_	Coiled coil	Avibacterium paragallinarum
D0QYN6	22.59	0.000000000003	ccbE	CcbE	_	Coiled coil	Avibacterium paragallinarum
Q9DUN0	31.97	0.00000001	_	Orf73	_	3D-structure	Human herpesvirus 8
Q91LX9	31.97	0.00000001	_	ORF73	_	_	Human herpesvirus 8
W5U981	24.81	0.00000001	_	Htt	_	_	Homo sapiens
Q76SB0	29.41	0.00000002	_	ORF 73	_	3D-structure	Human herpesvirus 8 type M
Q98148	29.41	0.00000002	_	Kaposi's sarcoma-associated herpes-like virus ORF73 homolog	_	3D-structure	Human herpesvirus 8
E5LC01	31.15	0.00000002	ORF73	LANA	_	_	Human herpesvirus 8
Q9DUM3	31.97	0.00000002	_	Latent nuclear antigen	_	3D-structure	Human herpesvirus 8
Q2KPA5	27.05	0.00000004	_	Clock	_	Coiled coil, DNA-binding, Repeat	Macrobrachium rosenbergii
D0QYL8	22.3	0.0000001	acbE	AcbE	_	Coiled coil	Avibacterium paragallinarum
B5SUM8	23.18	0.0000004	MED15	Mediator of RNA polymerase II transcription subunit 15	_	Activator, Coiled coil, Nucleus, Transcription, Transcription regulation	Hyla arborea

Genomic Object Editor: llo3158

PB id	Ident %	Eval	Gene	Description	EC number	Keywords	Organism
D3HMC2	100	0	_	Uncharacterized protein	_	Coiled coil, Reference proteome	Legionella longbeachae serogroup 1 (strain NSW150)
A0A1R4GY81	23.72	9,00E-54	_	Uncharacterized protein	_	Coiled coil, Reference proteome	Crenothrix polyspora
A0A1Y1IYW1	25.57	4,00E-50	_	Signal recognition particle receptor protein FtsY	_	Coiled coil, Receptor	Comamonas testosteroni
A0A1I4MS07	24.75	1,00E-49	_	Uncharacterized protein	_	Coiled coil	Nitrosomonas communis
A0A096GZV8	23.08	3,00E-45	_	Methyltransf_21 domain-containing protein	_	Coiled coil	Comamonas testosteroni
A0A1I3JE51	24.08	4,00E-41	_	Uncharacterized protein	_	Coiled coil	Nitrosomonas sp. Nm34
D6Z4R0	24.66	6,00E-41	_	Chromosome segregation ATPase-like protein	_	Coiled coil, Reference proteome	Desulfurivibrio alkaliphilus (strain DSM 19089 / UNIQEM U267 / AHT2)
A0A0W7Z290	25.41	8,00E-41	_	Uncharacterized protein	_	Coiled coil, Reference proteome	Comamonas kerstersii
A0A0F7KD18	25.04	1,00E-39	_	Uncharacterized protein	_	Coiled coil, Reference proteome	Nitrosomonas communis
A0A1F9IRE7	26.98	2,00E-38	_	Uncharacterized protein	_	Coiled coil	Deltaproteobacteria bacterium RIFCSPLOWO2_02_FULL_53_8
Q6X1Y7	22.33	4,00E-24	lepB	Effector protein B	_	Coiled coil, Transmembrane	Legionella pneumophila
Q5ZSM7	22.33	4,00E-24	lepB	LepB	_	Coiled coil, Transmembrane	Legionella pneumophila subsp. pneumophila (strain Philadelphia 1 / ATCC 33152 / DSM 7513)
Q7K5Q6	26.48	4,00E-23	maebl	Erythrocyte binding protein 3	_	Coiled coil, Signal	Plasmodium falciparum
Q8T5C7	26.48	4,00E-23	maebl	Chimeric erythrocyte-binding protein MAEBL	_	Coiled coil, Signal, Transmembrane	Plasmodium falciparum
Q7K5Q5	26.48	4,00E-23	maebl	Erythrocyte binding protein 2	_	Coiled coil, Signal	Plasmodium falciparum
E5LC01	20.78	1,00E-22	ORF73	LANA	_	_	Human herpesvirus 8
Q6A178	26.8	2,00E-22	mt1	Myosin tail 1 protein	_	Coiled coil	Cryptosporidium parvum
Q91LX9	21.05	3,00E-22	_	ORF73	_	_	Human herpesvirus 8
Q25893	26.88	5,00E-22	LSA-1	Liver stage antigen	_	Membrane, Transmembrane, Transmembrane helix	Plasmodium falciparum

Supplementary Table 1 List of the highest hits of the *L. longbeachae* capsule cluster genes against the NCBI database (Trembl).

O44934	23.21	9,00E-22	_	Myosin heavy chain isoform A	_	Actin-binding, ATP-binding, Coiled coil, Myosin, Nucleotide-binding	Doryteuthis pealeii
G1EIL6	20.49	1,00E-21	tnks1bp1	Tankyrase 1 binding protein 1	_	_	Danio rerio
Q9U0S6	23.82	1,00E-20	prm MHC	Pedal retractor muscle myosin heavy chain	_	Actin-binding, ATP-binding, Coiled coil, Myosin, Nucleotide-binding	Mytilus galloprovincialis
A0A140UGH3	23.68	1,00E-20	_	Myosin 2 heavy chain striated muscle	_	Actin-binding, ATP-binding, Coiled coil, Myosin, Nucleotide-binding	Aphonopelma

Genomic Object Editor: llo3159

PB id	Ident %	Eval	Gene	Description	EC number	Keywords	Organism
D3HMC3	100	0	_	Uncharacterized protein	_	Reference proteome	Legionella longbeachae serogroup 1 (strain NSW150)
A4BRT8	41.15	5,00E-131	_	Uncharacterized protein	_	Reference proteome	Nitrococcus mobilis Nb-231
A0A1H3CQ84	58.71	1,00E-69	_	Uncharacterized protein	_	Reference proteome	Roseicitreum antarcticum
A0A1X7A7H2	42.42	6,00E-66	_	Uncharacterized protein	_	Coiled coil, Reference proteome	Limimaricola soesokkakensis
A0A1Q4CSG6	55.22	1,00E-64	_	Uncharacterized protein	_	_	Rhodobacterales bacterium 65-51
A0A2E9GVE9	42.72	4,00E-48	_	Uncharacterized protein	_	_	Deltaproteobacteria bacterium
A0A2E6LM58	35.68	2,00E-30	_	Methyltransf_21 domain-containing protein	_	Coiled coil	Gammaproteobacteria bacterium
A0A0F7KKU9	35.58	2,00E-29	_	Methyltransf_21 domain-containing protein	_	Coiled coil, Reference proteome	Nitrosomonas communis
A0A1I3JDG0	35.58	2,00E-29	_	Methyltransferase, FkbM family	_	Coiled coil, Methyltransferase, Transferase	Nitrosomonas sp. Nm34
A0A1H2Q970	42.41	2,00E-27	_	Methyltransferase, FkbM family	_	Methyltransferase, Transferase	Nitrosomonas communis

Genomic Object Editor: llo3160

PB id	Ident %	Eval	Gene	Description	EC number	Keywords	Organism
D3HMC4	100	4,00E-170	_	Putative lipopolysaccharide core biosynthesis protein	_	Reference proteome	Legionella longbeachae serogroup 1 (strain NSW150)
A0A0P7WRL6	64.44	4,00E-105	_	Uncharacterized protein	_	_	Idiomarinaceae bacterium HL-53
A0A1Q4CSD3	53.78	2,00E-81	_	Uncharacterized protein	_	_	Rhodobacterales bacterium 65-51
A4BRT7	58.29	6,00E-81	_	Uncharacterized protein	_	Reference proteome	Nitrococcus mobilis Nb-231
A0A1H9L2A0	52.73	1,00E-79	_	Uncharacterized protein	_	Reference proteome	Litorimicrobium taeanense
A0A254QKC4	52.65	2,00E-79	_	Uncharacterized protein	_	Reference proteome	Phaeobacter sp. 22II1-1F12B
A0A1V0RJS1	54.13	5,00E-78	_	Uncharacterized protein	_	_	Roseovarius mucosus
K1XV28	52.27	6,00E-73	_	Uncharacterized protein	_	_	uncultured bacterium
A0YTV4	51.83	1,00E-72	_	TPR_REGION domain-containing protein	_	Coiled coil, Reference proteome, TPR repeat	Lyngbya sp. (strain PCC 8106)
Q9XC98	26.44	0.003	_	Lipopolysaccharide core biosynthesis protein RfaZ	_	Membrane, Transferase, Transmembrane, Transmembrane helix	Klebsiella pneumoniae
I7AU32	27.78	0.62	waaZ	3-deoxy-D-manno-oct-2-ulosonate III transferase WaaZ	2.4.99.15	Glycosyltransferase, Transferase	Escherichia coli

Genomic Object Editor: llo3161

PB id	Ident %	Eval	Gene	Description	EC number	Keywords	Organism
D3HMC5	100	0	_	Putative glycosyl transferase family 2	_	Reference proteome, Transferase	Legionella longbeachae serogroup 1 (strain NSW150)
Q07Z86	59.24	9,00E-140	_	Glycosyl transferase, family 2	_	Reference proteome, Transferase	Shewanella frigidimarina (strain NCIMB 400)
A0A0P8B481	60.88	1,00E-130	_	Family 2 glycosyltransferase	_	Transferase	Idiomarinaceae bacterium HL-53
A0A0F7M0S2	54.78	2,00E-122	_	Glycosyl transferase	_	Reference proteome, Transferase	Spongiibacter sp. IMCC21906
A0A1B7WX93	57	3,00E-119	_	Glycosyl transferase family 2	_	Transferase	Anabaena sp. MDT14b
A0A1G1H1U7	58.36	2,00E-117	_	Glycosyl transferase family 2	_	Transferase	Nitrospirae bacterium GWC2_57_9
A0A1X7A7Q4	51.1	7,00E-113	_	N-glycosyltransferase	_	Reference proteome, Transferase	Limimaricola soesokkakensis
A0A090SRU4	54.79	3,00E-112	_	Glyco_trans_2-like domain-containing protein	_	_	Vibrio maritimus
A0A1W9GAI3	54.61	1,00E-110	_	Glycosyl transferase family 2	_	Transferase	Nitrospira sp. SG-bin2
A0A1V0RJU4	50.47	3,00E-109	_	Putative glycosyl transferase	_	Transferase	Roseovarius mucosus
Q3ZK45	41.07	2,00E-20	epsG	EpsG	_	_	Lactococcus lactis
O66259	30.32	0.00000000000002	_	Glycosyltransferase	_	Transferase	Aggregatibacter actinomycetemcomitans
Q9XDQ0	30.67	0.0000000000001	ORF14	Putative glycosyltransferase	_	Transferase	Aggregatibacter actinomycetemcomitans
Q54129	31.22	0.00000000002	wbaN	Rhamnosyl transferase	_	Transferase	Salmonella enterica
Q9AQA9	30	0.00000000005	_	Putative rhamnosyltransferase	_	Transferase	Aggregatibacter actinomycetemcomitans
K4P2X6	33.33	0.0000003	wbyL	WbyL	_	_	Yersinia similis
Q8GNC0	30.56	0.000002	lgtA	N-acetylglucosamine glycosyltransferase	_	Transferase	Haemophilus ducreyi

Genomic Object Editor: llo3162

PB id	Ident %	Eval	Gene	Description	EC number	Keywords	Organism
D3HMC6	100	3,00E-161	_	Putative acylneuraminate cytidylyltransferase	_	Nucleotidyltransferase, Reference proteome, Transferase	Legionella longbeachae serogroup 1 (strain NSW150)
K2JYG6	70.45	2,00E-106	_	Acylneuraminate cytidylyltransferase	_	Nucleotidyltransferase, Reference proteome, Transferase	Gallaecimonas xiamenensis 3-C-1
A0A0F7M0J7	68.66	6,00E-104	_	CMP-N-acetylneuraminic acid synthetase	_	Reference proteome	Spongiibacter sp. IMCC21906
A0A0M1JD43	68.42	8,00E-101	_	Acylneuraminate cytidylyltransferase	_	Nucleotidyltransferase, Reference proteome, Transferase	Achromatium sp. WMS3
A0A0M1J3Y3	67.94	1,00E-99	_	Acylneuraminate cytidylyltransferase	_	Nucleotidyltransferase, Reference proteome, Transferase	Achromatium sp. WMS3
A0A011NPN0	65.9	1,00E-97	_	3-deoxy-manno-octulosonate cytidylyltransferase	_	Nucleotidyltransferase, Reference proteome, Transferase	Candidatus Accumulibacter sp. SK-11
A0A250KNV9	66.21	2,00E-97	_	Acylneuraminate cytidylyltransferase	_	Nucleotidyltransferase, Reference proteome, Transferase	Methylocaldum marinum

Supplementary Table 1 List of the highest hits of the *L. longbeachae* capsule cluster genes against the NCBI database (Trembl).

A0A1H9KWA5	63.27	2,00E-96	_	CMP-N-acetylneuraminic acid synthetase	_	Reference proteome	Litorimicrobium taeanense
A0A0N8KBF2	66.51	4,00E-96	neuA	N-acylneuraminate cytidylyltransferase NeuA	_	Nucleotidyltransferase, Transferase	Idiomarinaceae bacterium HL-53
A0A254QKC9	65.75	2,00E-95	_	Acylneuraminate cytidylyltransferase	_	Nucleotidyltransferase, Reference proteome, Transferase	Phaeobacter sp. 22II1-1F12B
Q933W2	23.64	0.0000001	neuA1	Acylneuraminate cytidylyltransferase	_	Nucleotidyltransferase, Transferase	Campylobacter jejuni
Q077S2	21.98	0.0000002	nnaC	Acylneuraminate cytidylyltransferase	2.7.7.43	Nucleotidyltransferase, Transferase	Escherichia coli

Genomic Object Editor: llo3163

PB id	Ident %	Eval	Gene	Description	EC number	Keywords	Organism
D3HMC7	100	0	_	Putative D-isomer specific 2-hydroxyacid dehydrogenase	_	NAD, Oxidoreductase, Reference proteome	Legionella longbeachae serogroup 1 (strain NSW150)
B8CL20	68.06	8,00E-152	_	D-isomer specific 2-hydroxyacid dehydrogenase, catalytic region, D-isomer specific 2-hydroxyacid dehydrogenase, NAD-binding	_	NAD, Oxidoreductase	Shewanella piezotolerans (strain WP3 / JCM 13877)
Q07Z88	67.2	1,00E-150	_	D-isomer specific 2-hydroxyacid dehydrogenase, NAD-binding	_	NAD, Oxidoreductase, Reference proteome	Shewanella frigidimarina (strain NCIMB 400)
A0A0P7ZKZ6	66.56	8,00E-147	serA	D-3-phosphoglycerate dehydrogenase	1.1.1.95	NAD, Oxidoreductase	Idiomarinaceae bacterium HL-53
A0A0C3MQY8	65.05	2,00E-143	_	Phosphoglycerate dehydrogenase	_	NAD, Oxidoreductase	Shewanella sp. cp20
A4BRT3	60.91	3,00E-133	_	Phosphoglycerate dehydrogenase	_	NAD, Oxidoreductase, Reference proteome	Nitrococcus mobilis Nb-231
A0A254QKD0	60.33	3,00E-126	_	Phosphoglycerate dehydrogenase	_	Oxidoreductase, Reference proteome	Phaeobacter sp. 22II1-1F12B
A0A0P1EMI7	58.55	1,00E-124	serA_2	D-3-phosphoglycerate dehydrogenase	1.1.1.95	Oxidoreductase, Reference proteome	Shimia marina
A0A1Q4CSG3	59.22	3,00E-124	_	Phosphoglycerate dehydrogenase	_	Oxidoreductase	Rhodobacterales bacterium 65-51
A0A1X7A7P1	58.17	1,00E-123	tkrA_2	Glyoxylate/hydroxypyruvate reductase B	1.1.1.79	Oxidoreductase, Pyruvate, Reference proteome	Limimaricola soesokkakensis
F8AEA4	35.64	8,00E-37	gyaR	Glyoxylate reductase	1.1.1.26	3D-structure, Cytoplasm, NAD, Oxidoreductase	Pyrococcus yayanosii (strain CH1 / JCM 16557)
Q2TL63	32.23	2,00E-32	_	D-3-phosphoglycerate dehydrogenase	1.1.1.95	Amino-acid biosynthesis, NAD, Oxidoreductase, Serine biosynthesis	Mesorhizobium ciceri
A8R0N0	30.55	1,00E-31	ApPGDH	D-3-phosphoglycerate dehydrogenase	1.1.1.95	Amino-acid biosynthesis, NAD, Oxidoreductase, Serine biosynthesis	Aphanothece halophytica
U5TVU1	32.22	4,00E-27	mcyI	McyI	_	Oxidoreductase	Nostoc sp. 152
G4XDR8	32.37	2,00E-26	ptxD	Phosphite dehydrogenase	_	3D-structure, Oxidoreductase	Ralstonia sp. 4506

Genomic Object Editor: llo3164

PB id	Ident %	Eval	Gene	Description	EC number	Keywords	Organism
Q8KWT4	31.47	5,00E-25	bacC	Dihydroanticapsin 7-dehydrogenase	1.1.1.385	Antibiotic biosynthesis, NAD, Oxidoreductase	Bacillus subtilis
Q9WYG0	32.51	1,00E-24	_	Uncharacterized oxidoreductase TM_0325	1.-.-.-	Oxidoreductase, Reference proteome	Thermotoga maritima (strain ATCC 43589 / MSB8 / DSM 3109 / JCM 10099)
P39640	30	2,00E-23	bacC	Dihydroanticapsin 7-dehydrogenase	1.1.1.385	3D-structure, Antibiotic biosynthesis, NAD, Oxidoreductase, Reference proteome	Bacillus subtilis (strain 168)
P50199	28.81	2,00E-23	gno	Gluconate 5-dehydrogenase	1.1.1.-	Carbohydrate metabolism, Cytoplasm, Direct protein sequencing, NADP, Oxidoreductase, Reference proteome	Gluconobacter oxydans (strain 621H)
Q56318	29.27	9,00E-23	_	Uncharacterized oxidoreductase TM_0019	1.-.-.-	NADP, Oxidoreductase, Reference proteome	Thermotoga maritima (strain ATCC 43589 / MSB8 / DSM 3109 / JCM 10099)
P40288	28.23	1,00E-21	_	Glucose 1-dehydrogenase	1.1.1.47	3D-structure, Direct protein sequencing, NADP, Oxidoreductase, Sporulation	Bacillus megaterium
Q51576	29.64	1,00E-21	_	Uncharacterized oxidoreductase PA3106	1.-.-.-	NADP, Oxidoreductase, Reference proteome	Pseudomonas aeruginosa (strain ATCC 15692 / DSM 22644 / CIP 104116 / JCM 14847 / LMG 12228 / 1C / PRS 101 / PAO1)
P39482	27.35	1,00E-21	gdhI	Glucose 1-dehydrogenase 1	1.1.1.47	Germination, NADP, Oxidoreductase, Sporulation	Bacillus megaterium
P46331	30.08	2,00E-21	yxbG	Uncharacterized oxidoreductase YxbG	1.-.-.-	NAD, Oxidoreductase, Reference proteome	Bacillus subtilis (strain 168)
Q92RN6	34.74	4,00E-21	galD	Probable galactose dehydrogenase GalD	1.1.1.-	NADP, Oxidoreductase, Reference proteome	Rhizobium meliloti (strain 1021)
P08074	30.36	6,00E-21	Cbr2	Carbonyl reductase	NADPH] 2	1.1.1.184	2455724, 7705352, 15489334, 8040004, 8999926, 21183079, 8805511
P05406	31.64	8,00E-21	fixR	Protein FixR	_	Nitrogen fixation, Oxidoreductase, Reference proteome	Bradyrhizobium diazoefficiens (strain JCM 10833 / BCRC 13528 / IAM 13628 / NBRC 14792 / USDA 110)
Q53882	28.96	8,00E-21	dauE	Aklaviketone reductase DauE	1.1.1.362	Antibiotic biosynthesis, NADP, Oxidoreductase	Streptomyces sp. (strain C5)

Genomic Object Editor: llo3165

PB id	Ident %	Eval	Gene	Description	EC number	Keywords	Organism
D3HMC9	100	0	_	Putative glycosyl transferase family 2	_	Reference proteome, Transferase	Legionella longbeachae serogroup 1 (strain NSW150)
A4BRT1	62.34	0	_	Uncharacterized protein	_	Reference proteome	Nitrococcus mobilis Nb-231
A0A1X7A785	58.81	0	_	Uncharacterized protein	_	Coiled coil, Reference proteome	Limimaricola soesokkakensis
A0A239Q0E7	54.37	0	_	Methyltransferase domain-containing protein	_	Coiled coil, Membrane, Methyltransferase, Reference proteome, Transferase, Transmembrane, Transmembrane helix	Amphiplicatus metriothermophilus
A0A2E7LM08	49.77	0	_	Glycosyl transferase family 2	_	Transferase	Dehalococcoidia bacterium
A0A1Q4CSM0	47.31	0	_	Uncharacterized protein	_	_	Rhodobacterales bacterium 65-51

Supplementary Table 1 List of the highest hits of the *L. longbeachae* capsule cluster genes against the NCBI database (Trembl).

A0A2M7GNM0	70.08	8,00E-129	_	Uncharacterized protein	_	Coiled coil, Membrane, Transmembrane, Transmembrane helix	Rhodobacterales bacterium CG15_BIG_FIL_POST_REV_8_21_14_020_59_13
K2KDH9	58.53	4,00E-99	_	Uncharacterized protein	_	Reference proteome	Gallaecimonas xiamenensis 3-C-1
B8CL24	59.92	1,00E-96	_	Uncharacterized protein	_	_	Shewanella piezotolerans (strain WP3 / JCM 13877)

Genomic Object Editor: llo3166

PB id	Ident %	Eval	Gene	Description	EC number	Keywords	Organism
D3HMD0	100	0	galE	UDP-glucose 4-epimerase	5.1.3.2	Carbohydrate metabolism, Isomerase, NAD, Reference proteome	Legionella longbeachae serogroup 1 (strain NSW150)
D3HMD6	63.44	1,00E-160	galE	UDP-glucose 4-epimerase	5.1.3.2	Carbohydrate metabolism, Isomerase, NAD, Reference proteome	Legionella longbeachae serogroup 1 (strain NSW150)
R4YS92	56.36	1,00E-131	galE	UDP-glucose 4-epimerase	5.1.3.2	Carbohydrate metabolism, Isomerase, NAD, Reference proteome	Oleispira antarctica RB-8
A0A2C4RG95	55.49	6,00E-129	galE	UDP-glucose 4-epimerase	5.1.3.2	Carbohydrate metabolism, Isomerase, NAD	Bacillus sp. AFS043905
A0A1H9KXM1	56.36	6,00E-127	_	UDP-glucose 4-epimerase	5.1.3.2	Carbohydrate metabolism, Isomerase, NAD	Butyrivibrio fibrisolvens
A0A1M6BZ40	56.06	1,00E-126	_	UDP-glucose 4-epimerase	5.1.3.2	Carbohydrate metabolism, Isomerase, NAD	Butyrivibrio fibrisolvens DSM 3071
A0A1H9ELV8	55.76	5,00E-126	_	UDP-glucose 4-epimerase	5.1.3.2	Carbohydrate metabolism, Isomerase, NAD, Reference proteome	Butyrivibrio sp. TB
A0A098L0R3	55.79	1,00E-124	_	UDP-glucose 4-epimerase	5.1.3.2	Carbohydrate metabolism, Coiled coil, Isomerase, NAD	Geobacillus thermoleovorans B23
A0A1G8VHA8	54.55	1,00E-124	_	UDP-glucose 4-epimerase	5.1.3.2	Carbohydrate metabolism, Coiled coil, Isomerase, NAD, Reference proteome	Lachnospiraceae bacterium G41
M5WMM1	53.5	1,00E-114	UGE	UDP-glucose 4-epimerase	5.1.3.-	Carbohydrate metabolism, Isomerase, NAD, Reference proteome	Prunus persica
Q58IJ6	53.33	3,00E-113	UGE1	UDP-glucose 4-epimerase	5.1.3.-	Carbohydrate metabolism, Coiled coil, Isomerase, NAD	Hordeum vulgare
Q9RP56	50.3	7,00E-113	galE	UDP-glucose 4-epimerase	5.1.3.2	Carbohydrate metabolism, Isomerase, NAD	Escherichia coli
Q58IJ5	50.45	4,00E-110	UGE2	UDP-glucose 4-epimerase	5.1.3.-	Carbohydrate metabolism, Isomerase, NAD	Hordeum vulgare
A0A2D0W0L9	49.24	1,00E-109	gne	UDP-glucose 4-epimerase	5.1.3.2	Carbohydrate metabolism, Isomerase, NAD	Escherichia fergusonii
Q2QD27	50	2,00E-109	gne	UDP-glucose 4-epimerase	5.1.3.2	Carbohydrate metabolism, Isomerase, NAD	Aeromonas hydrophila
M9VRS7	48.77	2,00E-109	galE2	UDP-glucose 4-epimerase	5.1.3.2	Carbohydrate metabolism, Isomerase, NAD	Streptococcus oralis

Genomic Object Editor: llo3167

PB id	Ident %	Eval	Gene	Description	EC number	Keywords	Organism
D3HMD1	100	0	gmd	GDP-mannose 4,6-dehydratase	4.2.1.47	Lyase, NADP, Reference proteome	Legionella longbeachae serogroup 1 (strain NSW150)
A0A024HQL7	76.09	0	bre-1	GDP-mannose 4,6-dehydratase	4.2.1.47	Lyase, NADP, Reference proteome	Pseudomonas knackmussii (strain DSM 6978 / LMG 23759 / B13)
A0A0A7EDA1	75.66	0	gmd	GDP-mannose 4,6-dehydratase	4.2.1.47	Lyase, NADP, Reference proteome	Pseudoalteromonas piratica
A0A0Q5FPZ0	75.22	0	gmd	GDP-mannose 4,6-dehydratase	4.2.1.47	Lyase, NADP	Pseudomonas sp. Leaf127
V9UXM5	74.34	0	gmd	GDP-mannose 4,6-dehydratase	4.2.1.47	Lyase, NADP	Pseudomonas monteilii SB3101
A0A177YY40	74.93	0	gmd_1	GDP-mannose 4,6-dehydratase	4.2.1.47	Lyase, NADP	Pseudomonas putida
A0A2J8I1D5	75.66	0	gmd	GDP-mannose 4,6-dehydratase	4.2.1.47	Lyase, NADP	Vibrio diazotrophicus
A0A2S5IDD8	74.93	0	gmd	GDP-mannose 4,6-dehydratase	4.2.1.47	Lyase, NADP	Pseudomonas aeruginosa
R1IXR6	75.37	0	gmd	GDP-mannose 4,6-dehydratase	4.2.1.47	Lyase, NADP	Grimontia indica
A0A2V4KC88	74.93	0	gmd	GDP-mannose 4,6-dehydratase	4.2.1.47	Lyase, NADP	Pseudomonas sp. MB-090624
A0A147GBA3	74.64	0	gmd	GDP-mannose 4,6-dehydratase	4.2.1.47	Lyase, NADP	Pseudomonas parafulva
F3KBD8	75.07	0	gmd	GDP-mannose 4,6-dehydratase	4.2.1.47	Lyase, NADP, Reference proteome	gamma proteobacterium IMCC2047
C8YZ33	74.49	0	gmd	GDP-mannose 4,6-dehydratase	4.2.1.47	Lyase, NADP	Escherichia coli
D2KWB3	73.47	0	gmd	GDP-mannose 4,6-dehydratase	4.2.1.47	Lyase, NADP	Pseudomonas savastanoi pv. glycinea
M4HNX2	71.51	6,00E-179	bceN	GDP-mannose 4,6-dehydratase	4.2.1.47	Lyase, NADP	Burkholderia cepacia
K4NNR8	53.26	8,00E-121	gmd	GDP-mannose 4,6-dehydratase	4.2.1.47	Lyase, NADP	Yersinia similis
Q9R966	51.63	4,00E-120	gmd	GDP-mannose 4,6-dehydratase	4.2.1.47	Lyase, NADP	Brucella melitensis
O85352	53.1	4,00E-120	gmd	GDP-mannose 4,6-dehydratase	4.2.1.47	Lyase, NADP	Caulobacter vibrioides
Q5ND85	52.26	8,00E-116	gmd	GDP-mannose 4,6-dehydratase	4.2.1.47	Lyase, NADP	Yersinia sp. A125 KOH2
A0A1B1R128	52.68	9,00E-116	GMD2	GDP-mannose 4,6-dehydratase	4.2.1.47	Lyase	Mortierella alpina
F1CLL6	51.15	1,00E-115	gmd	GDP-mannose 4,6-dehydratase	4.2.1.47	Lyase, NADP	Yersinia pseudotuberculosis
Q6T1X7	53.89	1,00E-115	gmd	GDP-mannose 4,6-dehydratase	4.2.1.47	Lyase, NADP	Aneurinibacillus thermoaerophilus

Genomic Object Editor: llo3168

PB id	Ident %	Eval	Gene	Description	EC number	Keywords	Organism
D3HMD2	100	0	_	Putative capsular polysaccharide biosynthesis protein	_	Coiled coil, Reference proteome	Legionella longbeachae serogroup 1 (strain NSW150)
A0A1Q4CWN3	47.39	6,00E-88	_	WcbI domain-containing protein	_	Coiled coil	Rhodobacterales bacterium 65-51
A0A2H4UTY7	28.2	5,00E-24	_	WcbI domain-containing protein	_	Reference proteome	Bodo saltans virus
A0A2N1DBJ2	26.25	0.0000000007	_	WcbI domain-containing protein	_	Reference proteome	Paraglaciecola sp. MB-3u-78
U7Q8X3	22.59	0.000000007	_	Nucleotide-diphospho-sugar transferase family protein	_	Transferase	Lyngbya aestuarii BL J
A0A1M7TIW0	28.49	0.0000009	_	WcbI domain-containing protein	_	Reference proteome	Desulfovibrio litoralis DSM 11393
Q93UJ4	25.22	0.0001	wcbI	WcbI	_	_	Burkholderia pseudomallei
Q63R74	24.78	0.0003	wcbI	Putative capsular polysaccharide biosynthesis protein	_	3D-structure, Reference proteome	Burkholderia pseudomallei (strain K96243)

Genomic Object Editor: llo3169

PB id	Ident %	Eval	Gene	Description	EC number	Keywords	Organism
D3HMD3	99.03	1,00E-66	_	Uncharacterized protein	_	Membrane, Reference protei	Legionella longbeachae serogroup 1 (strain NSW150)
NO MORE HITS!							

Genomic Object Editor: llo3170

PB id	Ident %	Eval	Gene	Description	EC number	Keywords	Organism
D3HMD4	100	0	_	SGL domain-containing protein	_	Reference proteome	Legionella longbeachae serogroup 1 (strain NSW150)

A0A1J8P5Y9	40.71	8,00E-59	_	Gluconolaconase	_	_	Candidatus Rickettsiella isopodorum
K2D0F4	38.67	4,00E-58	_	SMP-30/Gluconolaconase/LRE protein	_	_	uncultured bacterium
A0A1G0G7G2	38.67	4,00E-58	_	SGL domain-containing protein	_	_	Gammaproteobacteria bacterium RIFCSPHIGHO2_02_FULL_39_13
A8PKQ3	38.43	9,00E-58	_	SMP-30/Gluconolaconase/LRE domain protein	_	Reference proteome	Rickettsiella grylli
A0A1Y5DM83	34.91	9,00E-53	_	SGL domain-containing protein	_	_	Arcobacter sp. 31_11_sub10_T18
A9KEW0	34.96	1,00E-52	_	Gluconolactonase	3.1.1.17	Hydrolase	Coxiella burnetii (strain Dugway 5J108-111)
A0A2E5LWQ5	40.32	2,00E-52	_	Gluconolaconase	_	_	Dehalococcoidia bacterium
A0A1G0GPR6	37.8	2,00E-52	_	SGL domain-containing protein	_	_	Gammaproteobacteria bacterium RIFCSPHIGHO2_12_FULL_36_30
Q83AU0	34.59	2,00E-51	_	Gluconolactonase	3.1.1.17	Hydrolase, Reference proteome	Coxiella burnetii (strain RSA 493 / Nine Mile phase I)
A9CPS8	26.84	9,00E-19	GNL	Lactonase	_	_	Euglena gracilis
E7BDE8	26.95	2,00E-17	Dca	DCA protein	_	_	Drosophila guanche
Q8TA68	26.92	2,00E-17	H-LRE	Luciferin-regenerating enzyme	_	_	Aquatica lateralis
Q86DU5	24.53	6,00E-17	LRE	Luciferin-regenerating enzyme	_	_	Photinus pyralis

Genomic Object Editor: llo3171

PB id	Ident %	Eval	Gene	Description	EC number	Keywords	Organism
D3HMD5	100	0	galU	UTP--glucose-1-phosphate uridylyltransferase	2.7.7.9	Nucleotidyltransferase, Reference proteome, Transferase	Legionella longbeachae serogroup 1 (strain NSW150)
A0A1J4QD25	66.43	7,00E-133	_	UTP--glucose-1-phosphate uridylyltransferase	2.7.7.9	Nucleotidyltransferase, Reference proteome, Transferase	Oceanisphaera psychrotolerans
K6YSZ7	62.85	1,00E-132	galU	UTP--glucose-1-phosphate uridylyltransferase	2.7.7.9	Nucleotidyltransferase, Reference proteome, Transferase	Paraglaciecola arctica BSs20135
A0A233RFF0	66.55	2,00E-132	galU	UTP--glucose-1-phosphate uridylyltransferase	2.7.7.9	Nucleotidyltransferase, Reference proteome, Transferase	Oceanimonas doudoroffii
A0A135ZYV6	64.44	4,00E-132	_	UTP--glucose-1-phosphate uridylyltransferase	2.7.7.9	Nucleotidyltransferase, Reference proteome, Transferase	Paraglaciecola hydrolytica
R4YTW2	63.79	5,00E-132	galU	UTP--glucose-1-phosphate uridylyltransferase	2.7.7.9	Nucleotidyltransferase, Reference proteome, Transferase	Oleispira antarctica RB-8
A0A2S9VBQ5	65.23	6,00E-132	galU	UTP--glucose-1-phosphate uridylyltransferase	2.7.7.9	Nucleotidyltransferase, Transferase	Alteromonas alba
A0A2D5LH48	65.23	6,00E-132	galU	UTP--glucose-1-phosphate uridylyltransferase	2.7.7.9	Nucleotidyltransferase, Transferase	Alteromonas sp
A0A1E8FAQ7	65.34	9,00E-132	_	UTP--glucose-1-phosphate uridylyltransferase	2.7.7.9	Nucleotidyltransferase, Reference proteome, Transferase	Alteromonas lipolytica
A0A2D9S173	65.7	1,00E-131	galU	UTP--glucose-1-phosphate uridylyltransferase	2.7.7.9	Nucleotidyltransferase, Transferase	Alteromonadaceae bacterium
E1SP06	63.7	2,00E-131	_	UTP--glucose-1-phosphate uridylyltransferase	2.7.7.9	Nucleotidyltransferase, Reference proteome, Transferase	Ferrimonas balearica (strain DSM 9799 / CCM 4581 / KCTC 23876 / PAT)
C7FFE7	65.47	3,00E-128	_	UTP--glucose-1-phosphate uridylyltransferase	2.7.7.9	Nucleotidyltransferase, Transferase	Proteus mirabilis
A7KAV1	65	4,00E-127	_	UTP--glucose-1-phosphate uridylyltransferase	2.7.7.9	Nucleotidyltransferase, Transferase	Aeromonas hydrophila
O85215	62.37	2,00E-126	galU	UTP--glucose-1-phosphate uridylyltransferase	2.7.7.9	Nucleotidyltransferase, Transferase	Actinobacillus pleuropneumoniae
D4I3X5	63.7	6,00E-121	galU	UTP--glucose-1-phosphate uridylyltransferase	2.7.7.9	3D-structure, Nucleotidyltransferase, Transferase	Erwinia amylovora (strain CFBP1430)
Q70AL9	62.45	2,00E-120	galU	UTP--glucose-1-phosphate uridylyltransferase	2.7.7.9	Nucleotidyltransferase, Transferase	Yersinia enterocolitica
Q84BL0	60.07	8,00E-120	galU	UTP--glucose-1-phosphate uridylyltransferase	2.7.7.9	Nucleotidyltransferase, Transferase	Aeromonas hydrophila
Q848R8	59.04	1,00E-118	galU	UTP--glucose-1-phosphate uridylyltransferase	2.7.7.9	Nucleotidyltransferase, Transferase	Aeromonas hydrophila
Q70AL8	59.29	2,00E-111	galF	UTP--glucose-1-phosphate uridylyltransferase	2.7.7.9	Nucleotidyltransferase, Transferase	Yersinia enterocolitica
Q937X5	54.58	4,00E-97	galF	Alpha-D-glucosyl-1-phosphate uridylyltransferase	2.7.7.9	Nucleotidyltransferase, Transferase	Edwardsiella ictaluri
Q8GNG1	53.98	2,00E-96	galF	Alpha-D-glucosyl-1-phosphate uridylyltransferase	2.7.7.9	Lipopolysaccharide biosynthesis, Nucleotidyltransferase, Transferase	Escherichia coli

Genomic Object Editor: llo3172

PB id	Ident %	Eval	Gene	Description	EC number	Keywords	Organism
D3HMD6	100	0	galE	UDP-glucose 4-epimerase	5.1.3.2	Carbohydrate metabolism, Isomerase, NAD, Reference proteome	Legionella longbeachae serogroup 1 (strain NSW150)
D3HMD0	63.44	1,00E-160	galE	UDP-glucose 4-epimerase	5.1.3.2	Carbohydrate metabolism, Isomerase, NAD, Reference proteome	Legionella longbeachae serogroup 1 (strain NSW150)
R4YS92	60.42	6,00E-147	galE	UDP-glucose 4-epimerase	5.1.3.2	Carbohydrate metabolism, Isomerase, NAD, Reference proteome	Oleispira antarctica RB-8
A0A2E7E932	57.75	1,00E-131	galE	UDP-glucose 4-epimerase	5.1.3.2	Carbohydrate metabolism, Isomerase, NAD	Oceanospirillaceae bacterium
I0JQM5	53.89	2,00E-129	galE2	UDP-glucose 4-epimerase	5.1.3.2	Carbohydrate metabolism, Isomerase, NAD, Reference proteome	Halobacillus halophilus (strain ATCC 35676 / DSM 2266 / JCM 20832 / NBRC 102448/ NCIMB 2269)
A0A173ZI12	55.45	4,00E-129	galE_1	UDP-glucose 4-epimerase	5.1.3.2	Carbohydrate metabolism, Isomerase, NAD, Reference proteome	Clostridium ventriculi
A0A1H8C287	55.32	2,00E-127	_	UDP-glucose 4-epimerase	5.1.3.2	Carbohydrate metabolism, Isomerase, NAD	Paenisporosarcina quisquiliarum
A0A1I2KKT0	53.59	3,00E-127	_	UDP-glucose 4-epimerase	5.1.3.2	Carbohydrate metabolism, Isomerase, NAD	Halobacillus alkaliphilus
Q2QD27	54.27	1,00E-120	gne	UDP-glucose 4-epimerase	5.1.3.2	Carbohydrate metabolism, Isomerase, NAD	Aeromonas hydrophila
Q5JBH4	53.64	2,00E-116	galE	UDP-glucose 4-epimerase	5.1.3.2	Carbohydrate metabolism, Isomerase, NAD	Escherichia coli
Q4KXC7	53.03	3,00E-115	galE	UDP-glucose 4-epimerase	5.1.3.2	Carbohydrate metabolism, Isomerase, NAD	Escherichia coli
Q937X4	50.76	3,00E-114	galE	UDP-glucose 4-epimerase	5.1.3.2	Carbohydrate metabolism, Isomerase, NAD	Edwardsiella ictaluri

Supplementary Table 1 List of the highest hits of the *L. longbeachae* capsule cluster genes against the NCBI database (Trembl).

M9VRS7	49.25	6,00E-114	galE2	UDP-glucose 4-epimerase	5.1.3.2	Carbohydrate metabolism, Isomerase, NAD	Streptococcus oralis
A0A0F7YYT2	51.83	8,00E-114	gne1	UDP-glucose 4-epimerase	5.1.3.2	Carbohydrate metabolism, Isomerase, NAD	Acinetobacter baumannii AB5075
M9P0X5	52.44	2,00E-113	galE	UDP-glucose 4-epimerase	5.1.3.2	Carbohydrate metabolism, Isomerase, NAD	Providencia alcalifaciens
Q9X3S6	50.91	2,00E-113	galE	UDP-glucose 4-epimerase	5.1.3.2	Carbohydrate metabolism, Isomerase, NAD	Neisseria meningitidis
O54385	53.05	3,00E-113	galE	UDP-glucose 4-epimerase	5.1.3.2	Carbohydrate metabolism, Isomerase, NAD	Brucella abortus
Q9F8B2	51.66	4,00E-113	galE	UDP-glucose 4-epimerase	5.1.3.2	Carbohydrate metabolism, Isomerase, NAD	Moraxella catarrhalis

Genomic Object Editor: llo3173

PB id	Ident %	Eval	Gene	Description	EC number	Keywords	Organism
D3HMD7	99.69	0	fcl	GDP-L-fucose synthase	1.1.1.271	Isomerase, Multifunctional enzyme, NADP, Oxidoreductase, Reference proteome	Legionella longbeachae serogroup 1 (strain NSW150)
W8R8L8	69.62	3,00E-167	fcl	GDP-L-fucose synthase	1.1.1.271	Isomerase, Multifunctional enzyme, NADP, Oxidoreductase	Pseudomonas stutzeri
A0A2N8SP15	69.3	3,00E-167	fcl	GDP-L-fucose synthase	1.1.1.271	Isomerase, Multifunctional enzyme, NADP, Oxidoreductase	Pseudomonas stutzeri
A0A0D9AQH8	68.97	1,00E-166	fcl	GDP-L-fucose synthase	1.1.1.271	Isomerase, Multifunctional enzyme, NADP, Oxidoreductase	Pseudomonas stutzeri
A0A2E2GGE9	69.28	4,00E-166	fcl	GDP-L-fucose synthase	1.1.1.271	Isomerase, Multifunctional enzyme, NADP, Oxidoreductase	Pseudomonas sp
A0A2N1CMA6	68.97	4,00E-166	fcl	GDP-L-fucose synthase	1.1.1.271	Isomerase, Multifunctional enzyme, NADP, Oxidoreductase	Pseudomonas sp. Choline-3u-10
A0A172WSZ4	68.97	6,00E-166	fcl	GDP-L-fucose synthase	1.1.1.271	Isomerase, Multifunctional enzyme, NADP, Oxidoreductase	Pseudomonas stutzeri
A0A1M5PMK2	68.97	2,00E-165	fcl	GDP-L-fucose synthase	1.1.1.271	Isomerase, Multifunctional enzyme, NADP, Oxidoreductase	Pseudomonas xanthomarina DSM 18231
A0A2W5D1Z8	69.09	5,00E-165	fcl	GDP-L-fucose synthase	1.1.1.271	Isomerase, Multifunctional enzyme, NADP, Oxidoreductase	Pseudomonas kuykendallii
A0A078LSR6	68.44	7,00E-165	fcl	GDP-L-fucose synthase	1.1.1.271	Isomerase, Multifunctional enzyme, NADP, Oxidoreductase, Reference proteome	Pseudomonas saudiphocaensis
K4NRM2	66.98	1,00E-161	fcl	GDP-L-fucose synthase	1.1.1.271	Isomerase, Multifunctional enzyme, NADP, Oxidoreductase	Yersinia similis
Q56873	67.61	1,00E-158	fcl	GDP-L-fucose synthase	1.1.1.271	Isomerase, Multifunctional enzyme, NADP, Oxidoreductase	Yersinia enterocolitica
Q5ND84	64.89	1,00E-152	fcl	GDP-L-fucose synthase	1.1.1.271	Isomerase, Multifunctional enzyme, NADP, Oxidoreductase	Yersinia sp. A125 KOH2
Q4KXD6	64.38	2,00E-151	fcl	GDP-L-fucose synthase	1.1.1.271	Isomerase, Multifunctional enzyme, NADP, Oxidoreductase	Escherichia coli
Q5XL46	63.21	1,00E-150	wcaG	GDP-L-fucose synthase	1.1.1.271	Isomerase, Multifunctional enzyme, NADP, Oxidoreductase	Klebsiella pneumoniae
A5Y7V9	63.44	1,00E-149	fcl	GDP-L-fucose synthase	1.1.1.271	Isomerase, Multifunctional enzyme, NADP, Oxidoreductase, Reference proteome	Salmonella enterica subsp. enterica serovar Poona
Q9F7A3	62.81	8,00E-147	fcl	GDP-L-fucose synthase	1.1.1.271	Isomerase, Multifunctional enzyme, NADP, Oxidoreductase	Salmonella typhimurium

Genomic Object Editor: llo3174

PB id	Ident %	Eval	Gene	Description	EC number	Keywords	Organism
D3HMD8	100	0	_	Putative glycosyltransferase	_	Reference proteome, Transferase	Legionella longbeachae serogroup 1 (strain NSW150)
A0A011PZP5	50.53	1,00E-98	kfoC_1	Chondroitin polymerase	_	Transferase	Candidatus Accumulibacter sp. BA-92
A0A011PC78	53.31	9,00E-81	kfoC_1	Chondroitin polymerase	_	Reference proteome, Transferase	Candidatus Accumulibacter sp. SK-11
A0A011PMS9	51.82	3,00E-78	kfoC_1	Chondroitin polymerase	_	Reference proteome, Transferase	Candidatus Accumulibacter sp. SK-12
A4BRS0	48.95	5,00E-71	_	Glycosyltransferase	_	Reference proteome, Transferase	Nitrococcus mobilis Nb-231
A4CTD8	45.56	8,00E-67	_	Glycosyltransferase	_	Transferase	Synechococcus sp. (strain WH7805)
A0A1R4GZF7	54.37	1,00E-66	_	Glyco_trans_2-like domain-containing protein	_	Reference proteome	Crenothrix polyspora
L8BAN0	45.45	2,00E-66	_	Putative Glycosyltransferase	_	Transferase	Rubrivivax gelatinosus S1
D9PNG8	46.79	1,00E-61	_	Glycosyltransferase	_	Transferase	sediment metagenome
A0A2V6Q4G8	45.12	8,00E-60	_	Glycosyltransferase	_	Transferase	Candidatus Rokubacteria bacterium
Q6XQ52	29.11	2,00E-16	wbsK	Glycosyl transferase family 2	_	Transferase	Escherichia coli
D0QYM5	30.39	4,00E-16	acbD	AcbD	_	_	Avibacterium paragallinarum
D0QYL9	30.39	4,00E-16	acbD	AcbD	_	_	Avibacterium paragallinarum
Q9RFX0	36.43	5,00E-16	cps7H	Putative glycosyltransferase Cps7H	_	Transferase	Streptococcus suis
K4P2X6	29.74	0.000000000000002	wbyL	WbyL	_	_	Yersinia similis
F8RC13	34.13	0.000000000000005	wpaD	WpaD	_	_	Providencia alcalifaciens
Q8GMK1	32.17	0.00000000000001	wbsA	Glycosyl transferase family 2	_	Transferase	Escherichia coli
Q56869	28.95	0.0000000000001	wbcG	WbcG	_	_	Yersinia enterocolitica

A3F4D9	31.48	0.0000000000004	epsM	EpsM	_	Membrane, Plasmid, Transmembrane, Transmembrane helix	Lactococcus lactis subsp. cremoris
F1CLM1	33.06	0.0000000000008	wbzE	Putative glycosyl transferase	_	Transferase	Yersinia pseudotuberculosis

Genomic Object Editor: llo3175

PB id	Ident %	Eval	Gene	Description	EC number	Keywords	Organism
D3HMD9	100	0	_	Putative polysaccharide biosynthesis dehydrogenase/reductase protein	_	Membrane, Reference proteome, Transmembrane, Transmembrane helix	Legionella longbeachae serogroup 1 (strain NSW150)
A0A1G0WYL4	53.36	8,00E-88	_	Short-chain dehydrogenase	_	_	Legionellales bacterium RIFCSPHIGHO2_12_FULL_37_14
A0A1G0GZU1	53.91	5,00E-86	_	Short-chain dehydrogenase	_	Membrane, Transmembrane, Transmembrane helix	Gammaproteobacteria bacterium RIFCSPHIGHO2_12_FULL_40_19
A0A1G0G9X5	50.4	7,00E-77	_	Uncharacterized protein	_	Membrane, Transmembrane, Transmembrane helix	Gammaproteobacteria bacterium RIFCSPHIGHO2_02_FULL_39_13
K2BBG0	50.4	7,00E-77	_	Polysaccharide biosynthesis dehydrogenase/reductase protein	_	Membrane, Transmembrane, Transmembrane helix	uncultured bacterium
A0A2G6HQW6	48.21	1,00E-74	_	Short-chain dehydrogenase	_	Membrane, Transmembrane, Transmembrane helix	Thiothrix nivea
A0A2G6DK36	47.81	4,00E-72	_	Short-chain dehydrogenase	_	Membrane, Transmembrane, Transmembrane helix	Proteobacteria bacterium
A0A228K644	46.48	3,00E-71	_	SDR family oxidoreductase	_	_	Burkholderia sp. AU27893
A0A2S5DYT0	46.48	3,00E-71	_	KR domain-containing protein	_	_	Burkholderia contaminans
A0A103ZCF2	44.92	2,00E-70	_	Capsular biosynthesis protein	_	_	Burkholderia cepacia
A0A1V6KPJ8	45.31	3,00E-70	_	Capsular biosynthesis protein	_	_	Burkholderia cenocepacia
Q9KHD1	31.87	2,00E-19	_	Putative beta-ketoacyl reductase	_	_	Streptomyces griseus subsp. griseus
C6ZD46	30.89	9,00E-19	ydfG	NADP-dependent L-serine/L-allo-threonine dehydrogenase ydfG	1.1.1.-	Oxidoreductase	Legionella jamestowniensis
Q5EGQ6	32.02	5,00E-18	rkpH	RkpH	1.1.1.56	Oxidoreductase	Rhizobium fredii
B8XIJ9	28.72	9,00E-17	_	Ketoacyl-reductase like protein	_	_	Karlodinium veneficum
K7ZSN0	29.79	1,00E-16	lgnI	Gluconate 5-dehydrogenase	_	Reference proteome	Paracoccus laeviglucosivorans
Q8RR58	25.53	3,00E-16	acrM	Acyl coenzyme A reductase	_	Coiled coil	Acinetobacter sp. M-1
Q7LZT0	27.92	6,00E-16	_	3(or 17)beta-hydroxysteroid dehydrogenase I	1.1.1.51	Oxidoreductase	Anguilla japonica
Q9API9	29.69	6,00E-16	phaB	Acetoacetyl-CoA reductase	1.1.1.36	Oxidoreductase	Methylorubrum extorquens
Q6RH38	27.92	0.000000000000001	_	17b-hydroxysteroid dehydrogenase type I	_	Oxidoreductase	Anguilla japonica

Genomic Object Editor: llo3176

PB id	Ident %	Eval	Gene	Description	EC number	Keywords	Organism
D3HME0	100	0	_	Putative glycosyltransferase	2.4.1.-	Glycosyltransferase, Reference proteome, Transferase	Legionella longbeachae serogroup 1 (strain NSW150)
A0A2S5SX42	46.4	1,00E-100	_	Glycosyltransferase family 1 protein	_	Reference proteome, Transferase	Zhizhongheella caldifontis
H8FUS8	43.8	2,00E-94	_	Putative glycosyltransferase	2.4.-.-	Glycosyltransferase, Reference proteome, Transferase	Phaeospirillum molischianum DSM 120
A0A259BE26	43.86	1,00E-93	_	Glycos_transf_1 domain-containing protein	_	_	Halothiobacillus sp. 24-54-40
A0A0W0Z520	41.88	1,00E-91	_	Glycosyltransferase	2.4.1.-	Glycosyltransferase, Reference proteome, Transferase	Legionella shakespearei DSM 23087
A0A2N3B0J3	45.1	1,00E-85	_	Glycosyltransferase family 1 protein	_	Transferase	Alphaproteobacteria bacterium HGW-Alphaproteobacteria-7
A0A255Y7H4	38.96	8,00E-84	_	Glycos_transf_1 domain-containing protein	_	Reference proteome	Sandarakinorhabdus cyanobacteriorum
A0A1X1PB15	43.84	9,00E-81	_	Glycos_transf_1 domain-containing protein	_	Coiled coil	Burkholderia puraquae
T2N217	40.21	1,00E-80	_	Glycos_transf_1 domain-containing protein	_	_	Ralstonia sp. 5_2_56FAA
M4QN28	35.29	2,00E-64	wbdA	Mannosyltransferase	_	Glycosyltransferase, Transferase	Escherichia coli
Q47593	35.04	7,00E-64	mtfA	Mannosyltransferase A	_	Glycosyltransferase, Transferase	Escherichia coli
Q9LC66	35.04	7,00E-64	wbdA	Mannosyltransferase	_	Glycosyltransferase, Transferase	Klebsiella pneumoniae
Q9LC67	35.58	1,00E-62	wbdA	Mannosyltransferase	_	Glycosyltransferase, Transferase	Escherichia coli
C8YZ32	35.34	1,00E-60	wejI	WejI	_	_	Escherichia coli
O84908	32.57	1,00E-33	wbpX	Glycosyltransferase Gtf1	_	Transferase	Pseudomonas aeruginosa
Q93UK1	26.73	0.0000000000001	wcbB	WcbB	_	_	Burkholderia pseudomallei
M4M6T9	29.17	0.0000000006	_	Glycosyl transferase group 1	_	Transferase	Acidiphilium sp. PM
O84909	31.76	0.0000002	wbpY	Glycosyltransferase WbpY	_	Transferase	Pseudomonas aeruginosa
Q93UJ8	35.65	0.000005	wcbE	WcbE	_	_	Burkholderia pseudomallei
Q9RMT9	31.97	0.000005	wbdB	WbdB	_	_	Klebsiella pneumoniae

Genomic Object Editor: llo3177

PB id	Ident %	Eval	Gene	Description	EC number	Keywords	Organism
D3HME3	100	0	_	Uncharacterized protein	_	Membrane, Reference proteome, Transmembrane, Transmembrane helix	Legionella longbeachae serogroup 1 (strain NSW150)
A0A1G0GZV7	49.04	5,00E-130	_	Uncharacterized protein	_	_	Gammaproteobacteria bacterium RIFCSPHIGHO2_12_FULL_40_19
A0A1T4X2W9	36.1	5,00E-73	_	Glycosyltransferase family 28 C-terminal domain-containing protein	_	Coiled coil, Reference proteome, Transferase	Clostridium sp. USBA 49
U2CYI4	34	2,00E-68	_	Glycosyltransferase family 28 protein	_	Coiled coil, Reference proteome, Transferase	Clostridiales bacterium oral taxon 876 str. F0540
A0A1T4Y1N0	32.46	2,00E-60	_	Glycosyltransferase family 28 C-terminal domain-containing protein	_	Coiled coil, Reference proteome, Transferase	Caloramator quimbayensis
A0A2C6TA21	30.79	2,00E-51	_	Uncharacterized protein	_	Coiled coil	Nostoc linckia z16
A0A0C2V1S8	30.18	3,00E-49	_	Glyco_tran_28_C domain-containing protein	_	Reference proteome	Paenibacillus sp. VKM B-2647
A0A1I1D9I8	22.14	0.000000004	_	UDP-N-acetylglucosamine:LPS N-acetylglucosamine transferase	_	Glycosyltransferase, Reference proteome, Transferase	Brevinema andersonii

A0A1J4VM15	21.36	0.00000005	_	Uncharacterized protein	_	Glycosyltransferase, Membrane, Transferase, Transmembrane, Transmembrane helix	Candidatus Omnitrophica bacterium CG1_02_46_14
A0A1V4IVN9	20.83	0.0000001	ugtP	Processive diacylglycerol beta-glucosyltransferase	2.4.1.-	Glycosyltransferase, Reference proteome, Transferase	Clostridium chromiireducens

Genomic Object Editor: llo3178

PB id	Ident %	Eval	Gene	Description	EC number	Keywords	Organism
D3HME4	100	0	_	Putative aminotransferase	2.3.1.47	Acyltransferase, Aminotransferase, Pyridoxal phosphate	Legionella longbeachae serogroup 1 (strain NSW150)
A0A1G0WY90	63.45	0	_	8-amino-7-oxononanoate synthase	_	Pyridoxal phosphate	Legionellales bacterium RIFCSPHIGHO2_12_FULL_37_14
A0A157QUK5	61.34	0	wcbT	Polyketide synthase	2.3.1.-	Acyltransferase, Transferase	Bordetella trematum
A0A157SWX8	58.8	2,00E-180	wcbT	Polyketide synthase	2.3.1.-	Acyltransferase, Pyridoxal phosphate, Transferase	Bordetella ansorpii
A0A132F5H0	60.84	2,00E-179	_	8-amino-7-oxononanoate synthase	_	Pyridoxal phosphate	Burkholderia pseudomultivorans
A0A1I3G3G2	59.77	2,00E-179	_	8-amino-7-oxononanoate synthase	_	Pyridoxal phosphate	Collimonas sp. OK307
A0A1B4F580	61.07	3,00E-179	_	8-amino-7-oxononanoate synthase	_	Pyridoxal phosphate	Burkholderia sp. LA-2-3-30-S1-D2
A0A088UAL4	60.84	4,00E-179	_	Beta-eliminating lyase family protein	_	Lyase, Pyridoxal phosphate	Burkholderia cenocepacia
A0A2A4CCM4	60.61	1,00E-178	_	8-amino-7-oxononanoate synthase	_	Pyridoxal phosphate	Burkholderia sp. IDO3
Q5EGQ7	48.95	3,00E-134	rkpG	RkpG	2.3.1.29	Acyltransferase, Transferase	Rhizobium fredii
Q52936	51.62	3,00E-123	rkpG	Acyl-transferase	_	Transferase	Rhizobium meliloti
A7BFV7	33.33	6,00E-66	spt	Serine palmitoyltransferase	2.3.1.50	Acyltransferase, Pyridoxal phosphate, Transferase	Sphingobacterium spiritivorum
Q9AJN1	34.38	1,00E-65	bioF	8-amino-7-ketopelargonate synthase	2.3.1.47	Biotin biosynthesis, Pyridoxal phosphate, Transferase	Kurthia sp. 538-KA26
Q9AJM7	34.82	2,00E-65	bioFII	8-amino-7-ketopelargonate synthase	2.3.1.47	Biotin biosynthesis, Pyridoxal phosphate, Transferase	Kurthia sp. 538-KA26
A7BFV6	35.39	9,00E-65	spt	8-amino-7-oxononanoate synthase	2.3.1.47, 2.3.1.50	Acyltransferase, Pyridoxal phosphate, Transferase	Sphingobacterium multivorum
A7BFV8	32.14	2,00E-57	spt	Serine palmitoyltransferase	2.3.1.50	Acyltransferase, Pyridoxal phosphate, Transferase	Bacteriovorax stolpii
B2XR73	32.03	2,00E-56	LCB2	Serine C-palmitoyltransferase	2.3.1.50	Endoplasmic reticulum, Lipid metabolism, Membrane, Pyridoxal phosphate, Sphingolipid metabolism, Transferase, Transmembrane	Nicotiana benthamiana

Genomic Object Editor: llo3179

PB id	Ident %	Eval	Gene	Description	EC number	Keywords	Organism
D3HME5	100	0	rkpA	Malonyl CoA-acyl carrier protein transacylase	_	Multifunctional enzyme, NADP, Phosphopantetheine, Phosphoprotein, Reference proteome, Transferase	Legionella longbeachae serogroup 1 (strain NSW150)
A0A1G0H3Y5	48.92	0	_	Malonyl CoA-acyl carrier protein transacylase	_	Multifunctional enzyme, NADP, Phosphopantetheine, Phosphoprotein, Transferase	Gammaproteobacteria bacterium RIFCSPHIGHO2_12_FULL_38_11
A0A1G0GZW1	49.27	0	_	Malonyl CoA-acyl carrier protein transacylase	_	Multifunctional enzyme, NADP, Phosphopantetheine, Phosphoprotein, Transferase	Gammaproteobacteria bacterium RIFCSPHIGHO2_12_FULL_40_19
A0A1G0G9V3	47.94	0	_	Malonyl CoA-acyl carrier protein transacylase	_	Multifunctional enzyme, NADP, Phosphopantetheine, Phosphoprotein, Transferase	Gammaproteobacteria bacterium RIFCSPHIGHO2_02_FULL_39_13
K2C9B0	47.94	0	_	Carrier domain-containing protein	_	Multifunctional enzyme, NADP, Phosphopantetheine, Phosphoprotein, Transferase	uncultured bacterium
A0A1G0WYA8	46.27	0	_	Malonyl CoA-acyl carrier protein transacylase	_	Coiled coil, Multifunctional enzyme, NADP, Phosphopantetheine, Phosphoprotein, Transferase	Legionellales bacterium RIFCSPHIGHO2_12_FULL_37_14
A0A1T4X2T5	41.79	0	_	Malonyl CoA-acyl carrier protein transacylase	_	Coiled coil, Multifunctional enzyme, NADP, Phosphopantetheine, Phosphoprotein, Reference proteome, Transferase	Thiothrix eikelboomii
A0A2G6HQX1	41.42	0	_	Malonyl CoA-acyl carrier protein transacylase	_	Multifunctional enzyme, NADP, Phosphopantetheine, Phosphoprotein, Transferase	Thiothrix nivea
A0A2G6DKI5	41.28	0	_	Malonyl CoA-acyl carrier protein transacylase	_	Multifunctional enzyme, NADP, Phosphopantetheine, Phosphoprotein, Transferase	Proteobacteria bacterium
A0A1H9YTQ4	40.46	0	_	Malonyl CoA-acyl carrier protein transacylase	_	Multifunctional enzyme, NADP, Phosphopantetheine, Phosphoprotein, Transferase	Nitrosomonas europaea
Q82UT4	40.46	0	rkpA	Malonyl CoA-acyl carrier protein transacylase	_	Multifunctional enzyme, NADP, Phosphopantetheine, Phosphoprotein, Reference proteome, Transferase	Nitrosomonas europaea (strain ATCC 19718 / CIP 103999 / KCTC 2705 / NBRC 14298)

A0A238XMI4	40.34	0	_	Malonyl CoA-acyl carrier protein transacylase	_	Multifunctional enzyme, NADP, Phosphopantetheine, Phosphoprotein, Transferase	Methylobacillus rhizosphaerae
Q6E7K0	30.12	0	_	3-hydroxyacyl-CoA dehydrogenase	1.1.1.35	3D-structure, Coiled coil, Multifunctional enzyme, NADP, Nucleotide-binding, Phosphopantetheine, Phosphoprotein, Transferase	Lyngbya majuscula
A0A0A0WDX2	31.31	0	puwB	PuwB	_	Coiled coil, Multifunctional enzyme, NADP, Phosphopantetheine, Phosphoprotein, Transferase	Cylindrospermum alatosporum CCALA 988
F4Y426	28.41	0	_	CurJ	_	3D-structure, Phosphopantetheine, Phosphoprotein, Reference proteome, Transferase	Moorea producens 3L
Q6DNE3	28.41	0	curJ	CurJ	_	3D-structure, Phosphopantetheine, Phosphoprotein, Transferase	Lyngbya majuscula
Q5EGQ8	31.94	0	rkpA	Malonyl CoA-acyl carrier protein transacylase	_	Multifunctional enzyme, Phosphopantetheine, Phosphoprotein, Transferase	Rhizobium fredii
Q6ZY03	44.68	0	pks4	Polyketide synthase I	_	Phosphopantetheine, Phosphoprotein, Transferase	uncultured bacterium
A0A1B3TNB2	35.66	0	hapB	Malonyl CoA-acyl carrier protein transacylase	_	Coiled coil, Multifunctional enzyme, NADP, Phosphopantetheine, Phosphoprotein, Transferase	Byssovorax cruenta
Q9KIZ7	34.07	0	epoD	Malonyl CoA-acyl carrier protein transacylase	_	Coiled coil, Multifunctional enzyme, Phosphopantetheine, Phosphoprotein, Transferase	Sorangium cellulosum
Q3KRU5	25.72	0	pksX1	PKSX1	_	Acyltransferase, Coiled coil, Methyltransferase, Multifunctional enzyme, NADP, Oxidoreductase, Phosphopantetheine, Phosphoprotein, Transferase	Xylaria sp. BCC 1067
Q8RJY0	34.6	0	stiG	Malonyl CoA-acyl carrier protein transacylase	_	Coiled coil, Phosphopantetheine, Phosphoprotein, Transferase	Stigmatella aurantiaca
Q93TW8	34.15	0	mxaD	Malonyl CoA-acyl carrier protein transacylase	_	Coiled coil, Phosphopantetheine, Phosphoprotein, Transferase	Stigmatella aurantiaca

Genomic Object Editor: llo3180

PB id	Ident %	Eval	Gene	Description	EC number	Keywords	Organism
D3HME6	100	0	capI	Protein capI	_	Reference proteome	Legionella longbeachae serogroup 1 (strain NSW150)
D5BMZ1	63.17	3,00E-152	_	Putative nucleotide sugar epimerase	5.1.3.-	Isomerase, Reference proteome	Puniceispirillum marinum (strain IMCC1322)
A0A149VYE0	65.06	4,00E-152	rfbB_2	dTDP-glucose 4,6-dehydratase	4.2.1.46	Lyase, Reference proteome	Ferrovum sp. Z-31
A0A1Q8YJ37	63.64	2,00E-151	_	NAD-dependent epimerase/dehydratase	_	Reference proteome	Rhodoferax antarcticus ANT.BR
A0A1F4K1Q8	61.76	4,00E-150	_	Protein CapI	_	_	Burkholderiales bacterium RIFCSPLOWO2_12_FULL_61_40
A0A2E1QD17	60.66	4,00E-148	_	Epimerase domain-containing protein	_	_	Euryarchaeota archaeon
A0A1F9MKL1	62.54	2,00E-146	_	Capsular biosynthesis protein CpsI	_	_	Deltaproteobacteria bacterium RIFOXYD12_FULL_53_23
A0A1H7HPY1	60.36	2,00E-146	_	UDP-glucuronate 4-epimerase	_	_	Roseateles sp. YR242
A0A1F9KZI9	62.84	1,00E-145	_	Epimerase domain-containing protein	_	_	Deltaproteobacteria bacterium RIFOXYD12_FULL_56_24
Q6U8B8	59.16	1,00E-138	_	Putative nucleotide sugar epimerase	_	_	Raoultella terrigena
Q9RP53	57.66	6,00E-134	wbnF	NAD-dependent epimerase	4.2.1.46	Lyase	Escherichia coli
Q4GY28	55.52	5,00E-127	wbnF	UDP-sugar epimerase	_	_	Erwinia amylovora
Q6URR1	54.63	9,00E-127	nse	Putative epimerase	_	_	Xenorhabdus nematophila
O68979	55.22	3,00E-121	wcvA	Nucleotide sugar epimerase	_	_	Vibrio vulnificus
Q56626	53.45	1,00E-116	_	Nucleotide sugar epimerase	_	_	Vibrio cholerae O139
P96481	48.18	5,00E-100	cap1J	Putative epimerase	_	_	Streptococcus pneumoniae
Q70PA0	44.11	3,00E-90	_	Epimerase domain-containing protein	_	_	Melittangium lichenicola
I1VCA9	45.24	6,00E-90	GAE1	UDP-D-glucuronate 4-epimerase 1	_	_	Arabidopsis thaliana
Q6K9M5	44.97	1,00E-88	_	Os02g0791500 protein	_	Membrane, Reference proteome, Transmembrane, Transmembrane helix	Oryza sativa subsp. japonica
