# Supplementary material for: The unique Legionella longbeachae capsule favors intracellular replication and immune evasion
Source: PLoS Pathog. 2024 Sep 11;20(9):e1012534. doi: 10.1371/journal.ppat.1012534 (PMC11419355; doi:10.1371/journal.ppat.1012534)
Supplement: S3 Table — (PDF) [file ppat.1012534.s003.pdf]

Supplementary Table 3 List of genes up-or downregulated in E vs. PE phase in the *Llo* WT compared to the capsule mutant.

Genes up- or downregulated in E Phase (WT is the reference)				
**Gene**	**Gene name**	**log2 FC**	**padj**	**Putative function**
llo0333	--	2.036	3,32E-07	Glycine transporter
llo0471	--	-2.248	0,020045124	Unknown protein
llo0616	*groES*	-2.105	0,002161758	Co-chaperonin with GroEL
llo0666	--	-2.053	0,011655845	Uroporphyrin-III C-methyltransferase
llo0686	*asnA*	-2.958	0,000245832	Asparagine synthetase A
llo1148	--	-2.196	1,92E-05	Secreted endonuclease
llo1188	--	2.392	3,11E-08	Unknown protein
llo1391	--	-2.093	3,07E-10	Putative Thiol:disulfide interchange protein
llo1421	--	-2.626	0,001866207	Putative fatty acid desaturase
llo1641	--	-2.307	2,32E-05	Unknown protein, weakly similar to eukyotic proteins
llo2353b	--	3.107	2,08E-05	Arginine ABC transporter
llo2487	--	2.008	4,21E-07	Unknown protein
llo2505	*dnaK*	-2.18	0,001354314	Chaperone protein
llo2810	--	2.142	0,001841016	Truncated transposase
llo2873	--	-2.26	0,006984806	LvrB homolog
llo2874	*csrA*	-2.808	7,46E-05	Translational regulator
llo3149	*bexD*	-3.508	1,96E-51	CPS export protein
llo3150	*ctrD*	-6.947	2,97E-95	CPS export, ATP-binding protein
llo3151	*ctrC*	-5.952	3,50E-50	CPS export, IM binding protein
llo3266	--	2.141	1,79E-09	Unknown protein
llo3308	--	2.221	1,89E-05	Putative endoglucanase
llo3444	--	2.546	1,83E-08	Coiled-coil protein
LEGLO_2011	*llo4038*	-2.036	0,001680686	Unknown protein
LEGLO_2385	*llo4052*	2.797	0,071249112	Unknown protein
LEGLO_2621	*llo4063*	2.709	2,23E-11	Unknown protein
LEGLO_2873	*llo4071*	-3.731	7,79E-07	Transposase
LEGLO_5S_1	*rRNA 5S*	-5.284	7,37E-09	--
LEGLO_5S_2	*rRNA 5S*	-5.259	7,76E-09	--
LEGLO_5S_3	*rRNA 5S*	-5.21	6,16E-09	--
LEGLO_5S_4	*rRNA 5S*	-5.279	7,37E-09	--
LEGLO_tRNA19	*tRNA-Glu*	-2.508	2,62E-08	--
LEGLO_tRNA24	*tRNA-Val*	-2.13	0,002206954	--
LEGLO_tRNA27	*tRNA-Asp*	-2.753	1,55E-07	--
LEGLO_tRNA28	*tRNA-Ser*	-2.744	1,00E-11	--
LEGLO_tRNA32	*tRNA-Lys*	-2.073	0,002388847	--
LEGLO_tRNA36	*tRNA-Ser*	-3.552	4,97E-10	--
LEGLO_tRNA44	*tRNA-Arg*	-2.288	4,97E-10	--
**Genes up- or downregulated in PE Phase (WT is the reference)**				
**Gene**	**Gene name**	**log2 FC**	**padj**	**Putative function**
llo0455	--	-2.46	0,01866126	Unknown protein
llo0591	--	-2.346	0,009072375	Hypothetical protein
llo1676b	--	-2.423	0,039328406	Transposase
llo2373	--	2.687	0,01866126	Putative periplasmic binding protein
llo2945	--	-2.181	0,021237205	Unknown protein
llo3150	*ctrD*	-5.117	9,28E-26	CPS export, ATP-binding protein
llo3151	*ctrC*	-6.678	6,71E-23	CPS export, IM binding protein
LEGLO_2219	*llo4044*	-2.108	0,004311215	Unknown protein
LEGLO_2873	*llo4071*	-3.678	1,97E-05	Transposase
LEGLO_tRNA11	*tRNA-Gly*	-3.076	6,26E-06	--
LEGLO_tRNA13	*tRNA-Leu*	-2.084	0,034102743	--
LEGLO_tRNA19	*tRNA-Glu*	-2.234	0,04047741	--
LEGLO_tRNA20	*tRNA-Pro*	-4.088	6,26E-06	--
LEGLO_tRNA21	*tRNA-Arg*	-2.533	7,24E-05	--
LEGLO_tRNA22	*tRNA-His*	-2.848	0,001197106	--
LEGLO_tRNA24	*tRNA-Val*	-3.081	5,33E-05	--
LEGLO_tRNA25	*tRNA-Asp*	-2.985	0,000570294	--
LEGLO_tRNA26	*tRNA-Val*	-2.966	7,93E-06	--
LEGLO_tRNA27	*tRNA-Asp*	-3.278	0,000491633	--
LEGLO_tRNA28	*tRNA-Ser*	-2.703	0,000570294	--
LEGLO_tRNA29	*tRNA-Arg*	-2.53	0,001861708	--
LEGLO_tRNA32	*tRNA-Lys*	-2.997	3,08E-06	--
LEGLO_tRNA36	*tRNA-Ser*	-5.31	2,47E-05	--
LEGLO_tRNA40	*tRNA-Asn*	-4.144	1,62E-07	--
LEGLO_tRNA44	*tRNA-Arg*	-3.541	9,34E-06	--
LEGLO_tRNA6	*tRNA-Thr*	-2.396	0,021237205	--

Color code	p-value adj.
(blue)	p>0,01
(grey)	0,01≤p<0,05

Color code	p-value adj.
(blue)	p>0,01
(grey)	0,01≤p<0,05
