# Supplementary figures and images for: The unique Legionella longbeachae capsule favors intracellular replication and immune evasion
Source: PLoS Pathog. 2024 Sep 11;20(9):e1012534. doi: 10.1371/journal.ppat.1012534 (PMC11419355; doi:10.1371/journal.ppat.1012534)

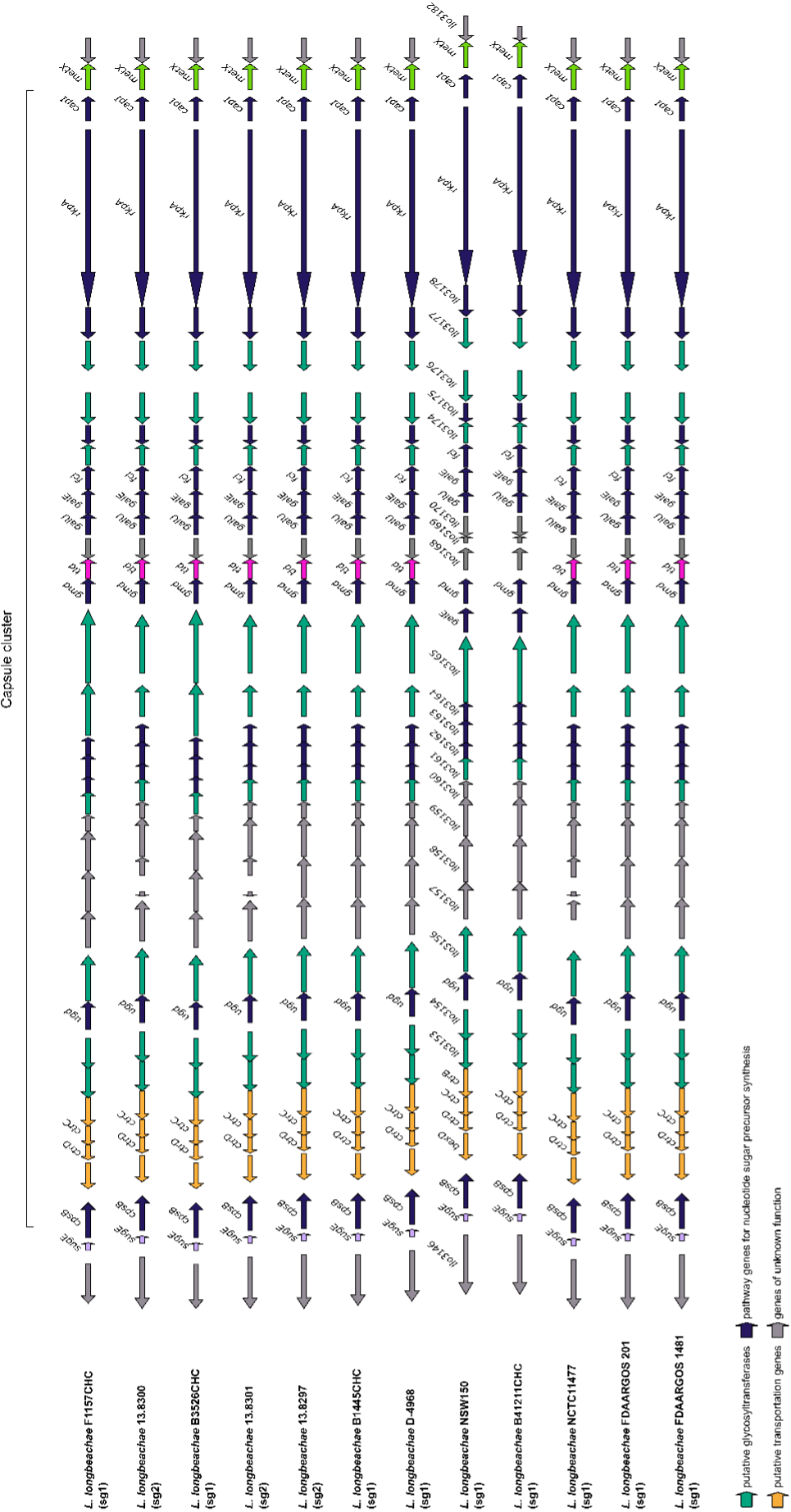

Supplement: S1 Fig — Visualization of the gene content of the cluster capsule in serogroup 1 and serogroup 2 Legionella longbeachae strains using GeneSpy [107]. Genes coding for the capsule plus two flanking genes on either side are represented. To obtain homogeneous and comparable annotations, all the genome sequences were reannotated using PROKKA. Based on the new annotation files, gene names and biochemical functions are used to infer families, and a color is attributed for each one. (TIF) [file ppat.1012534.s004.tif]

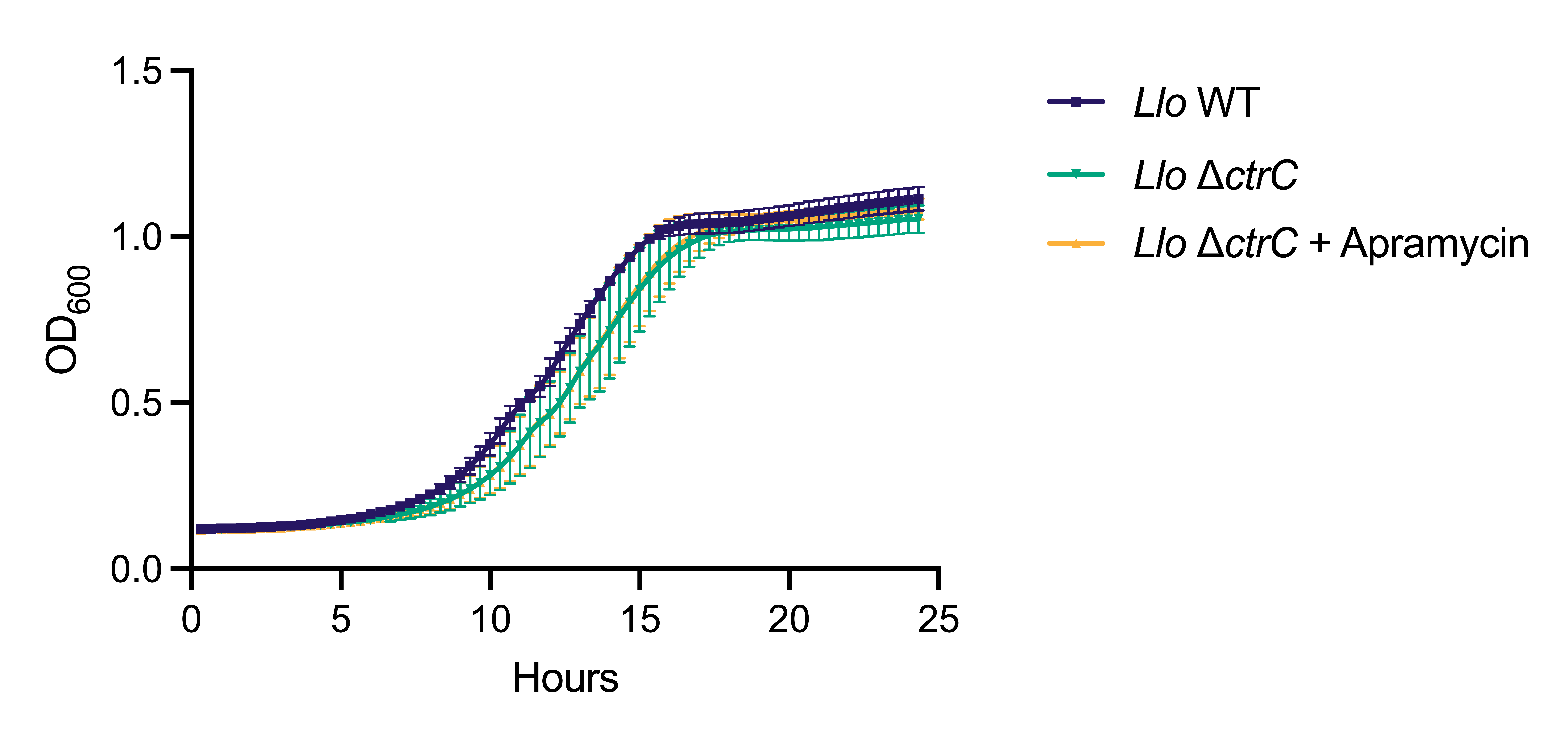

Supplement: S2 Fig — Llo WT or ΔctrC ± apramycin were grown in BYE medium at 37°C and OD600 was followed using a BioTek Synergy Plate Reader 2. Data show means ± SD of n = 3 independent experiments. (TIF) [file ppat.1012534.s005.tif]

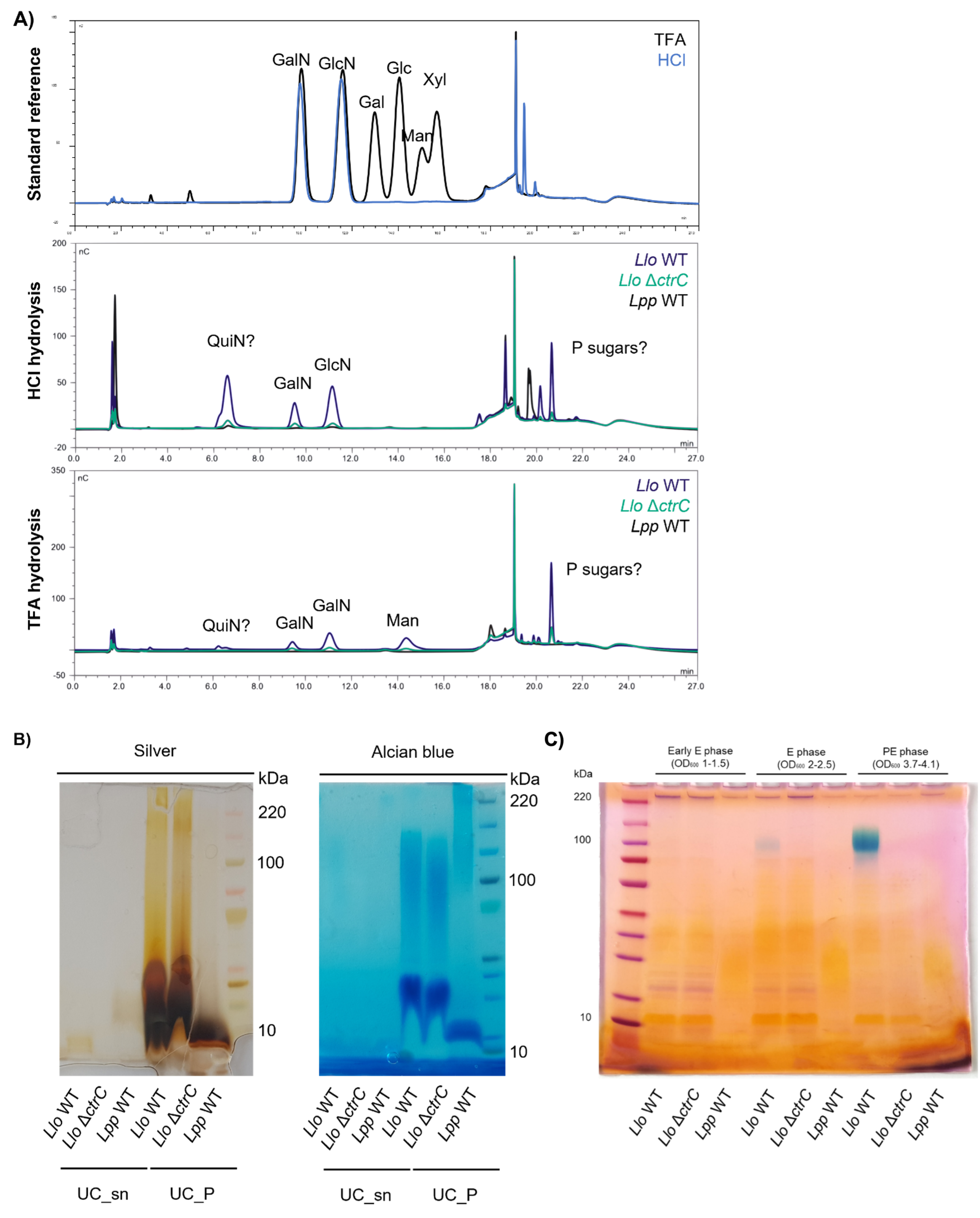

Supplement: S4 Fig — A) Elution profiles of High-Performance Anion Exchange Chromatography. Top graph, standard reference hydrolyzed with TFA. Middle and bottom panel, bacterial phenol extracts hydrolyzed with HCl and TFA. Blue line, Llo WT; green line, Llo ΔctrC; black line, Lpp WT. B) SDS gel electrophoresis of phenol extracts stained with silver nitrate or Alcian blue. C) Gel electrophoresis of extracts from Llo WT, ΔctrC or Lpp WT grown at 20°C in BYE to different optical densities and stained with Stains-all dye. QuiN, quinovosamine; GalN, galactosamine; GlcN, glucosamine; Gal, galactose; Glc, glucose; Man, mannose; Xyl, xylose; UC_sn, supernatants after ultracentrifugation; UC_P, pellets after ultracentrifugation; E, exponential; PE, post-exponential. (TIF) [file ppat.1012534.s007.tif]

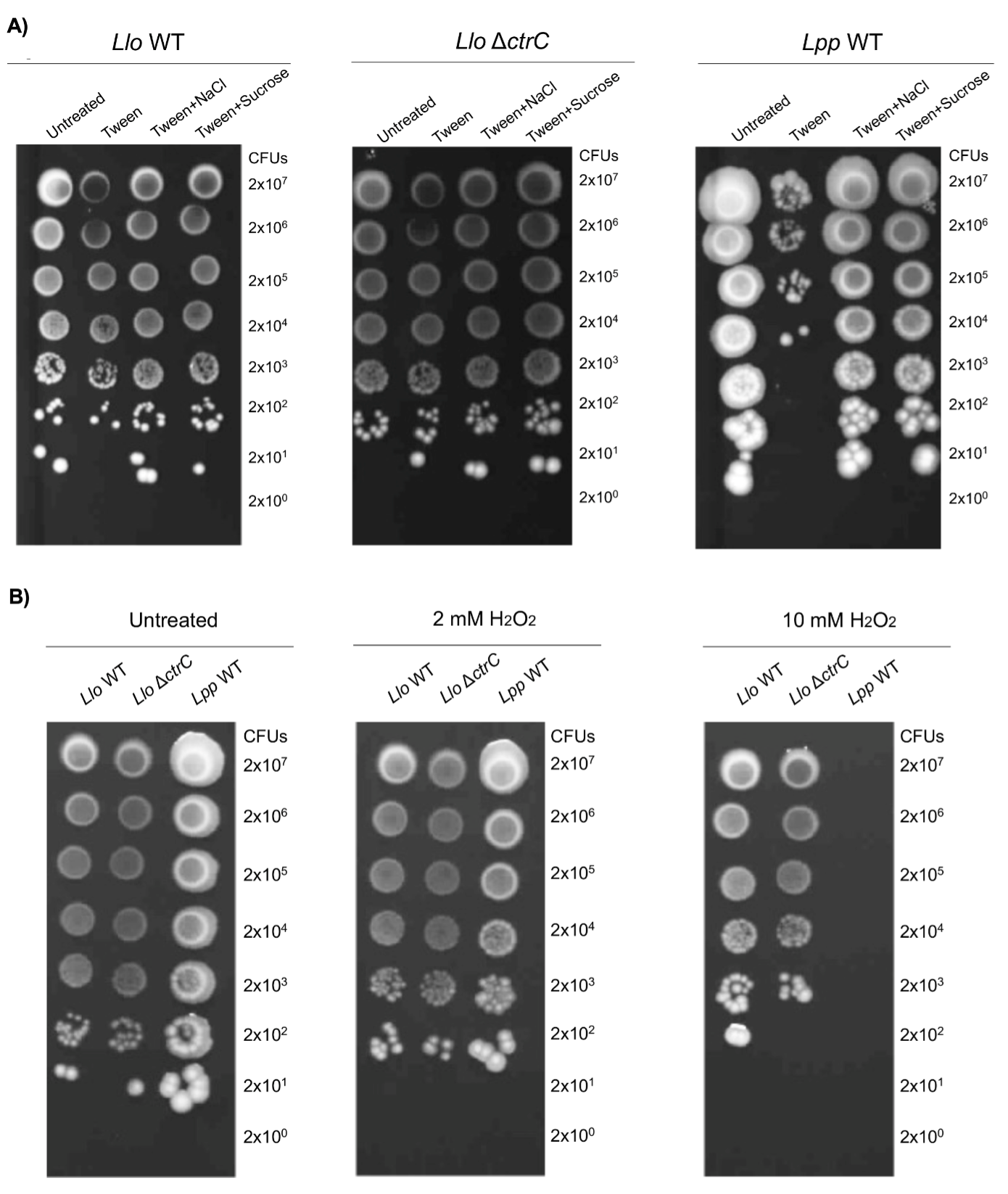

Supplement: S5 Fig — Bacteria were grown to PE phase (OD600 3.7–4.2) in BYE medium for each experiment. A) Treatment with Tween-20 ± 300 mM NaCl or 300 mM sucrose. B) Treatment with 2 mM or 10 mM H2O2. Representative images of n = 2 independent experiments. (TIF) [file ppat.1012534.s008.tif]

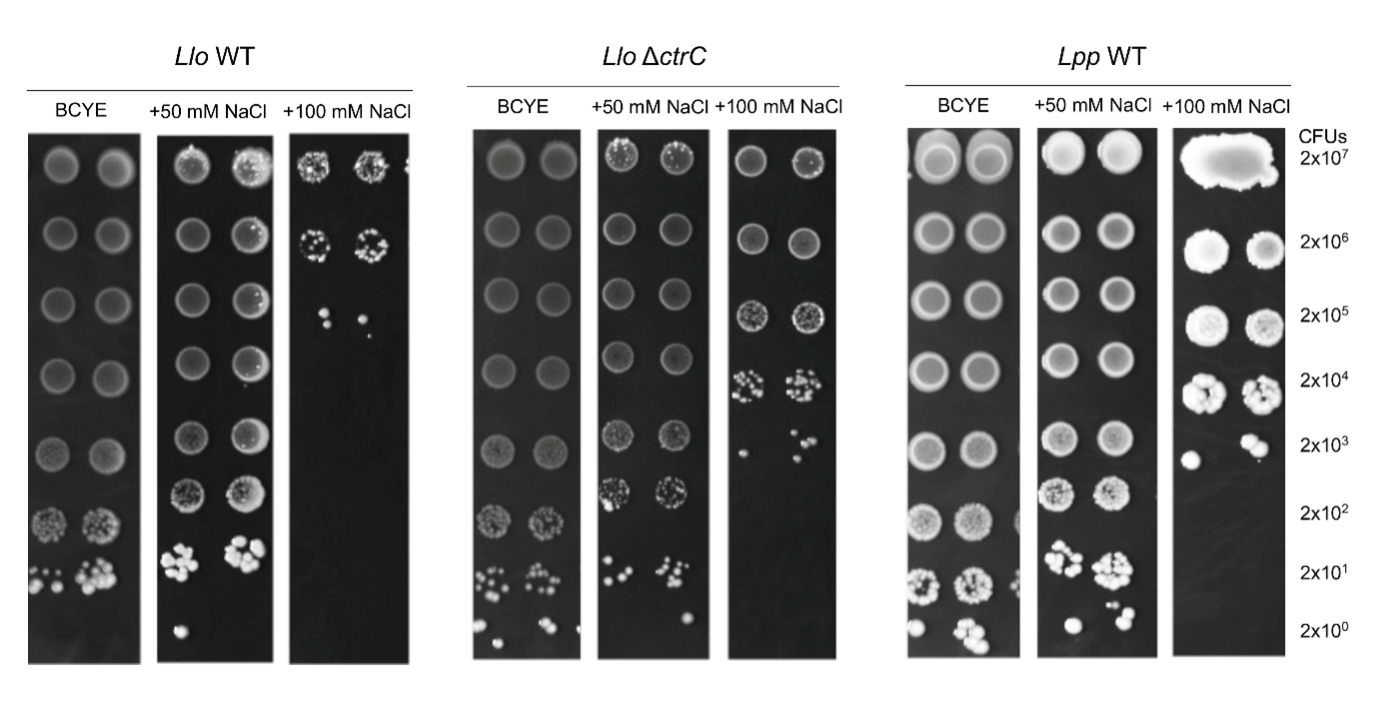

Supplement: S6 Fig — Cells were grown to PE phase (OD600 3.7–4.2) in BYE medium and spotted onto BCYE plates ± NaCl. Representative images of n = 3 independent experiments. (TIF) [file ppat.1012534.s009.tif]

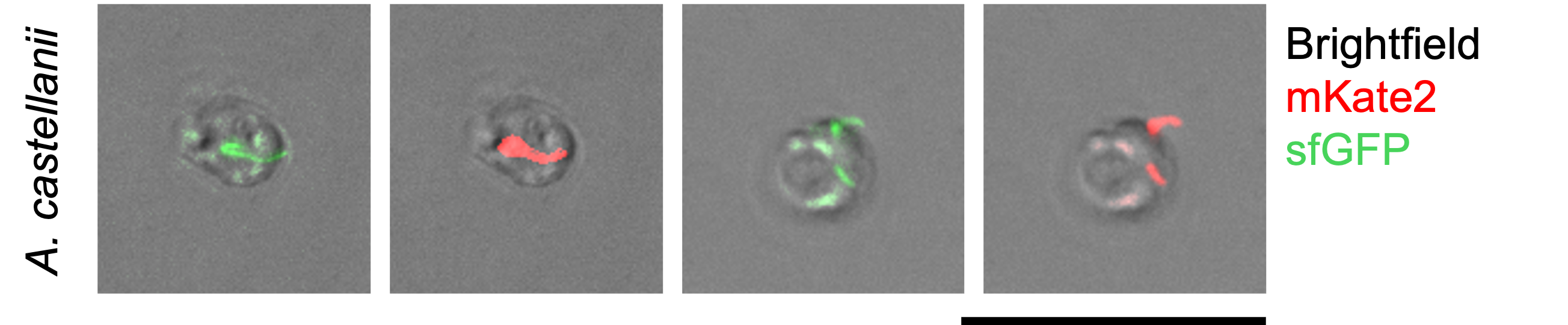

Supplement: S8 Fig — A. castellanii was infected with Llo WT bacteria harboring the dual reporter plasmid (pSS017) at MOI 10 and 25°C for 1 hour. Cells were imaged 48 hours post-infection using an EVOS inverted digital microscope. Scale bar = 50 μm. (TIF) [file ppat.1012534.s011.tif]

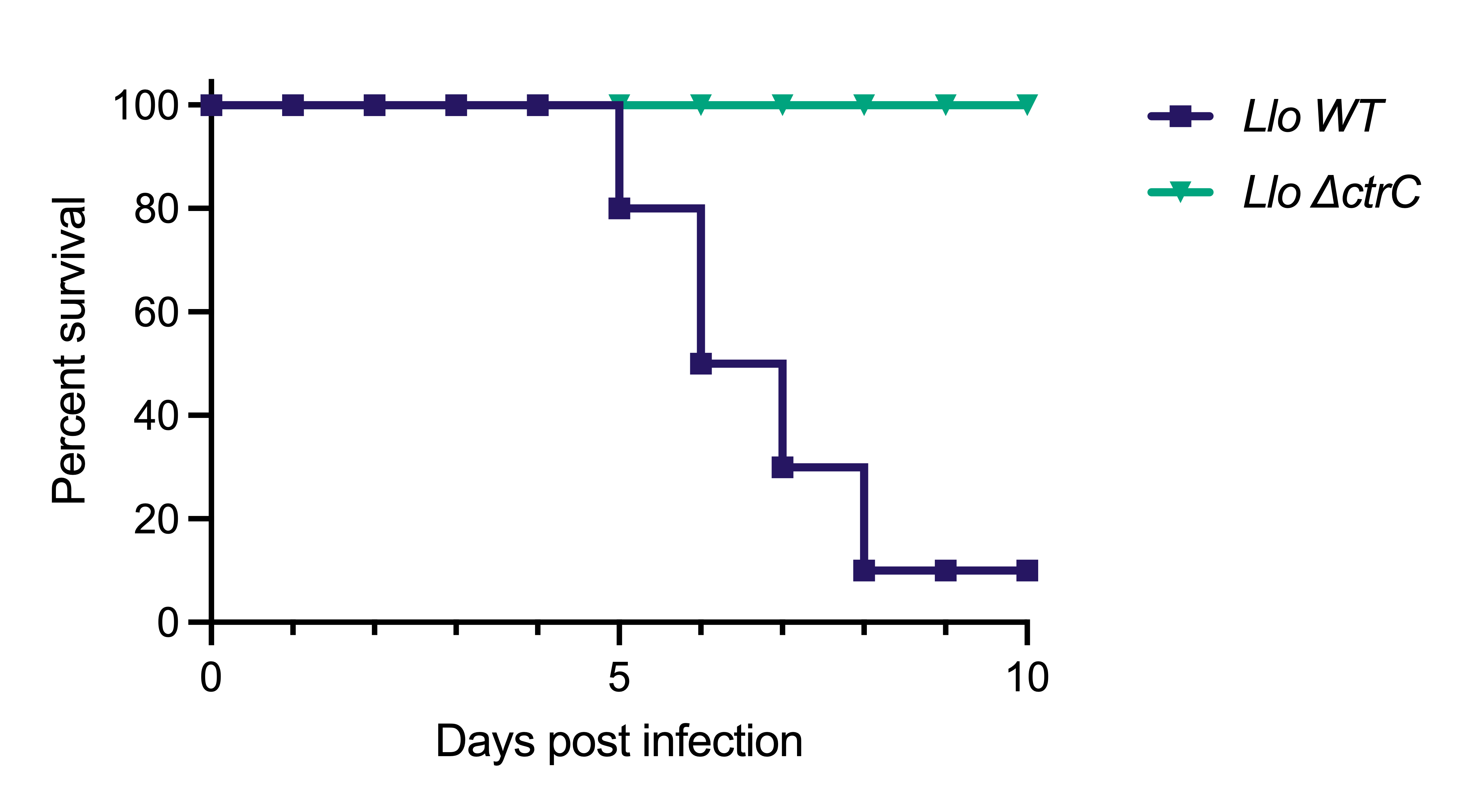

Supplement: S9 Fig — A) Female C57BL/6 mice were infected with 106 bacteria and survival was monitored over ten days. Survival was monitored for 9 mice per group. (TIF) [file ppat.1012534.s012.tif]

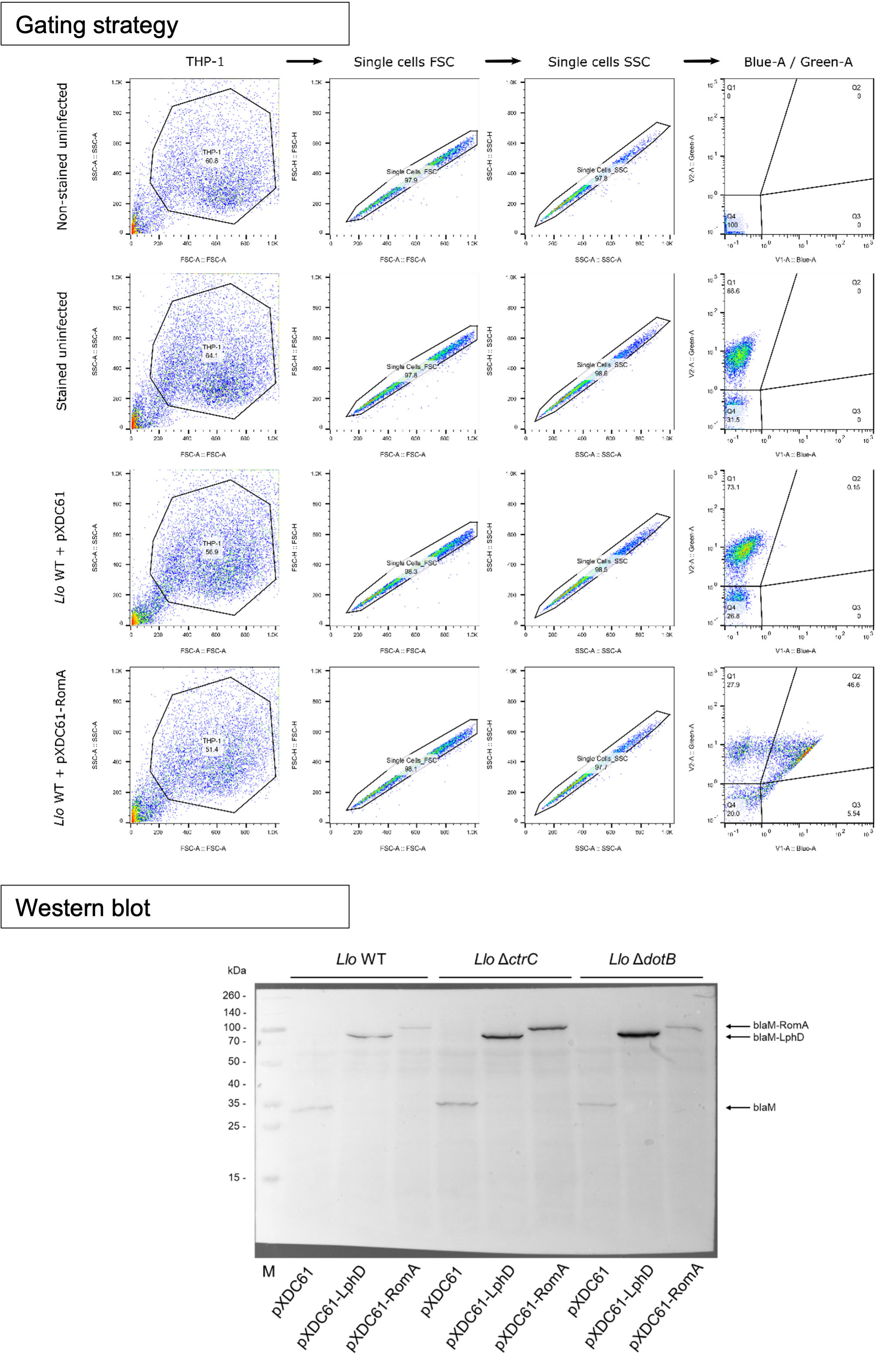

Supplement: S10 Fig — A) Gating strategy for flow cytometry. Cells were gated on THP-1 cells, and single cells were gated by FSC and SSC. Within the single cell population, gates for the signal showing the blue channel and the green channel were set based on non-infected stained cells. B) Original Western Blot showing the expression of BlaM-constructs with the known T4SS effectors LphD and RomA in L. longbeachae. (TIF) [file ppat.1012534.s013.tif]

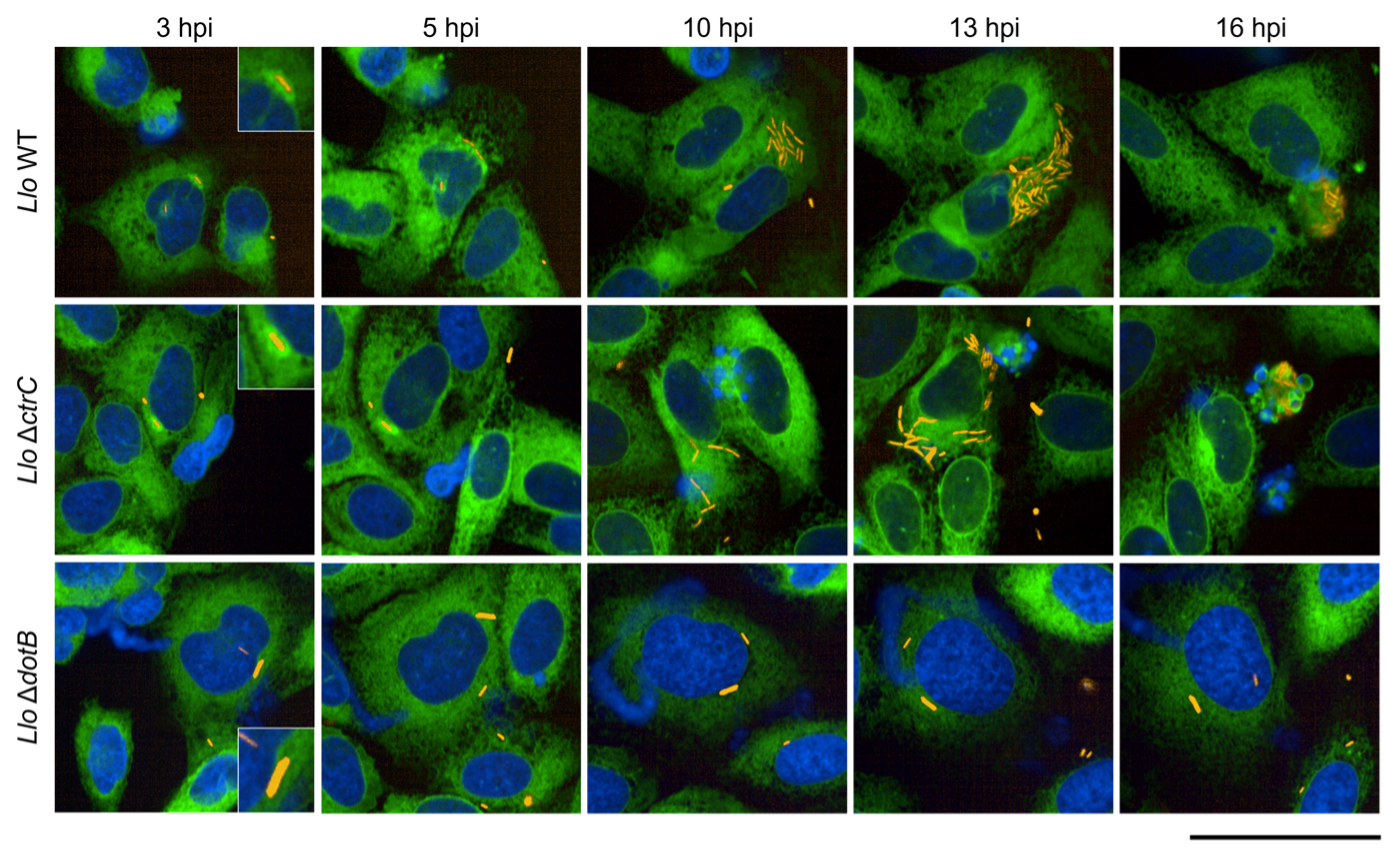

Supplement: S11 Fig — A) U2OS cells constitutively expressing Sec61-GFP were infected with Llo WT, ΔctrC or ΔdotB at MOI 100 and LCV formation was followed over time. Note that both Llo WT and ΔctrC recruit Sec61b early in infection (white arrows), but not ΔdotB (see image inlets). Green: Sec61b-GFP; yellow: L. longbeachae; blue: Hoechst dye. Representative images of n = 3 independent experiments. Scale bar = 50 μm. (TIF) [file ppat.1012534.s014.tif]

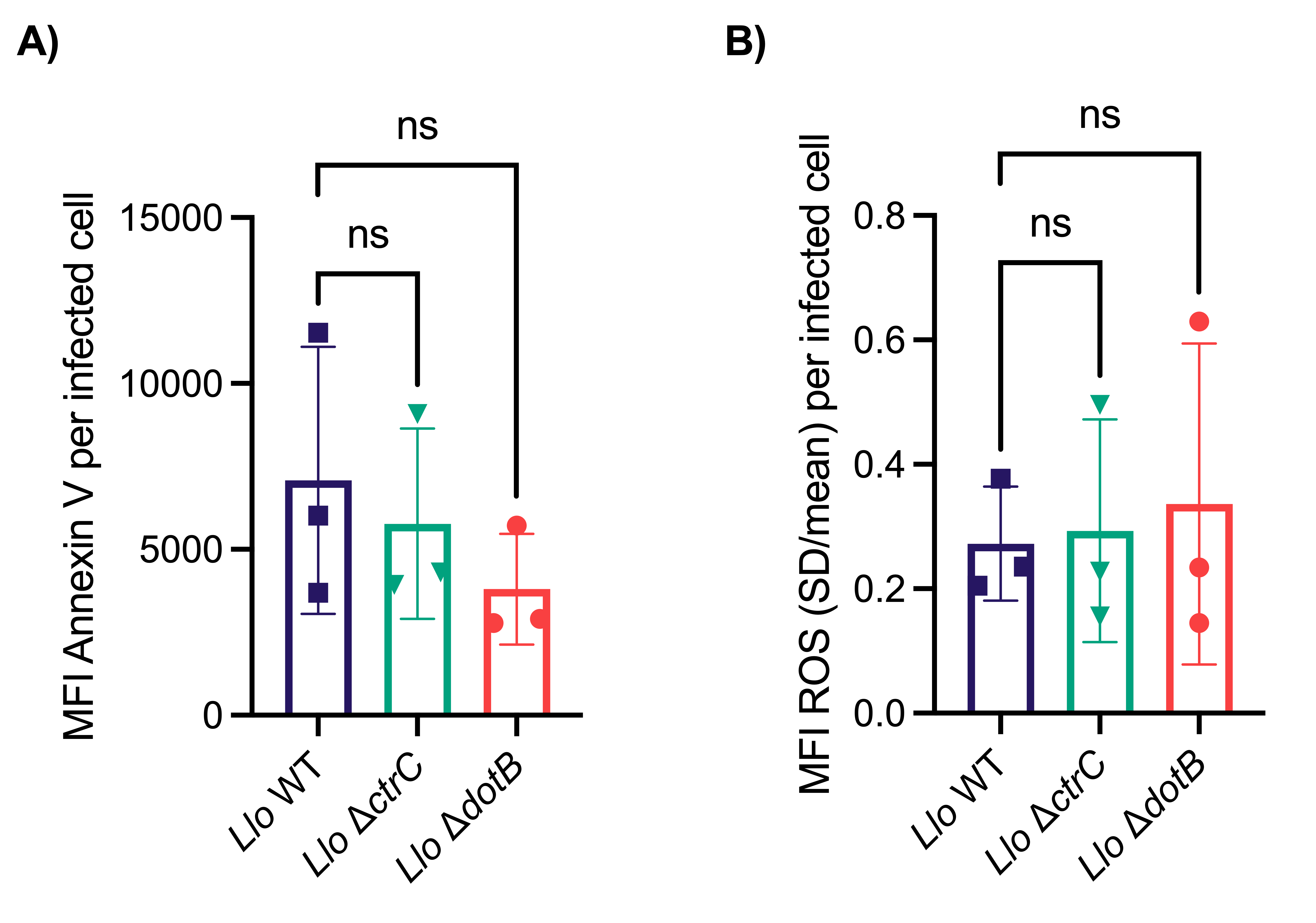

Supplement: S12 Fig — A) hMDMs were infected at MOI 10 with Llo WT, ΔctrC or ΔdotB and labelled with Annexin V dye for early apoptosis induction at 20 hours post-infection. B) hMDMs were infected at MOI 10 with Llo WT, ΔctrC or ΔdotB and labelled with CellROX dye for ROS production at 20 hours post-infection. Data show mean fluorescence intensity (MFI) ±SD of infected cells for n = 3 independent experiments. Statistical analysis was performed by one-way ANOVA. ns, non-significant. (TIF) [file ppat.1012534.s015.tif]

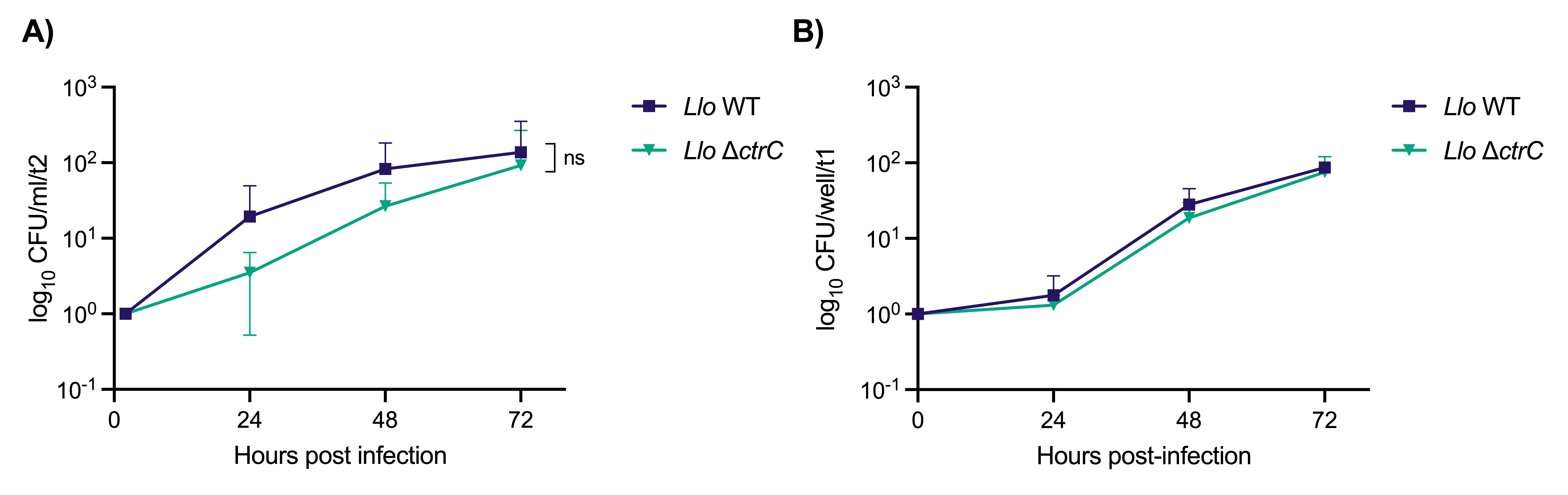

Supplement: S13 Fig — A) Differentiated THP-1 cells were infected at MOI 10 for 1 hour and treated with gentamycin to kill extracellular bacteria. CFUs were plated every 24 hours and normalized to the input control. Data show means ± SD of n = 6 independent experiments B) Bone marrow-derived macrophages (BMDMs) were infected at MOI 10 and CFUs plated every 24 hours normalized to the input control. Data show means ± SD of n = 3 independent experiments. (TIF) [file ppat.1012534.s016.tif]

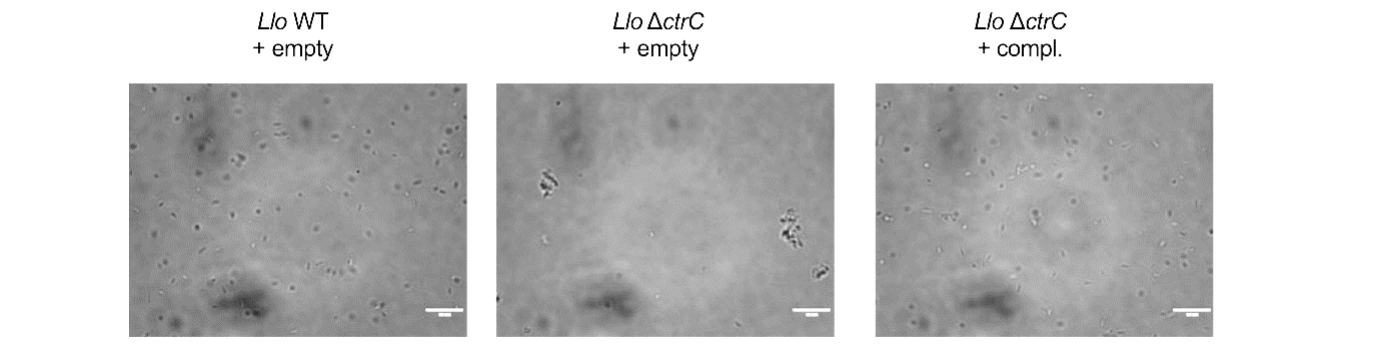

Supplement: S14 Fig — Bacteria were grown to PE phase (OD600 3.7–4.2), washed, and treated with 1 mg/ml yeast mannan for 15 minutes. Scale bar = 10 μm. (TIF) [file ppat.1012534.s017.tif]
