# Supplementary material for: The unique Legionella longbeachae capsule favors intracellular replication and immune evasion
Source: PLoS Pathog. 2024 Sep 11;20(9):e1012534. doi: 10.1371/journal.ppat.1012534 (PMC11419355; doi:10.1371/journal.ppat.1012534)
Supplement: Attachment — Submitted filename: Point by point answers.pdf [file ppat.1012534.s023.pdf]

Part I - Summary
Please use this section to discuss strengths/weaknesses of study, novelty/significance, general execution and scholarship.

Reviewer #1: The manuscript by Schmidt et al. reports the characterization of Legionella longbeachae capsule as a new virulence factor that confers L. longbeachae its virulence in vivo in mice and its natural host Acanthamoeba castellani. The manuscript's content is interesting, but the structure of the text impairs the reading. Paragraphs should be rearranged (e.g. salt stress in the middle of the in vivo/in cellulo experiments; paragraphs with eukaryotic cells, then mice, then on cells again). Mice experiments are the strongest and should represent the last part of the results. The title "The unique Legionella longbeachae capsule favors intracellular replication and immune evasion" doesn't seem to fully fit the manuscript as no strong evidence is presented (no experiment on LCV, microscopy imaging on replication/evasion, no difference on THP-1/BMDM replication in FigS8).

As suggested by the reviewer, we have restructured the text, the salt stress paragraph has been moved up as suggested, and all mouse data for virulence are together and all cellular data, too. However, we do not agree that the title is not fully fitting the manuscript as replication data had been supplied and, in addition, we added now several new replication experiments, microscopy images, and cytokine measurements in different hosts and in human primary cells that further support the title. Please see the point-by-point answers below for detailed explanation.

Reviewer #2: The paper convincingly demonstrates that in contrast to Legionella pneumophila (Lp), Legioenlla longbeachae (Llo) expresses a highly anionic capsule and that capsule formation can be disrupted by a mutation in the gene ctrC. The manuscript furthermore convincingly demonstrates that a ctrC mutant is less virulent in both amoebal and murine hosts. In case of mammalian cell infection, cell culture data implicates a role for capsule in phagocytosis avoidance and limitation of a proinflammatory cytokine response. Therefore, evasion of an immune response may underlie the virulence potential of the capsule in mice. However, whether these cell culture observations are linked to what happens during an in vivo infection was not further explored.
The MS provides evidence that a ctrC mutant has a growth defect within amoeba but not within human monocytic THP1 cells or murine bone marrow derived macrophages – these data thus suggest that loss of virulence in amoeba vs the mammalian host is mechanistically distinct. The MS does not provide any mechanistic model to account for the virulence defect in amoeba. The latter question warrants at least at some further discussion.
The data presentation/ figure design/ data organization could be substantially improved to render figures more reader-friendly and the content of the entire MS more readily digestible. Not all experimental designs include all the expected controls and/or meaningful timepoints. Despite these various shortcomings, this work constitutes an intriguing and important contribution to the Legionella literature.

Reviewer #3: This study characterises a polysaccharide capsule produced by Legionella longbeachae (Llo). Capsules are unprecedented across the Legionella genus and so the discovery of this genetic locus and the composition of the capsule is of interest. The authors identified that capsule expression is growth phase

dependent and that the capule comprises highly anionic polysaccharide, although they did not define the carbohydrate composition. A capsule mutant (delta ctrC) showed defects in intracellular replication in Acanthamoeba and reduced virulence in a mouse infection model, which defines the capsule as a major virulence factor of Llo. This adds to knowledge of the role of capsules in bacterial virulence although this has been shown previously for different pathogens. I think the authors need to reevaluate some of the conclusions as outlined in my comments.

Part II – Major Issues: Key Experiments Required for Acceptance
Please use this section to detail the key new experiments or modifications of existing experiments that should be absolutely required to validate study conclusions.

Generally, there should be no more than 3 such required experiments or major modifications for a "Major Revision" recommendation. If more than 3 experiments are necessary to validate the study conclusions, then you are encouraged to recommend "Reject".

Line numbers for the Figure legends correspond to the line numbers in the “tracked changes file” as we did not add the figures nor the supplemental figures in the clean file.

Reviewer #1: The authors stated that the capsule is expressed in eukaryotic hosts, but there is no explanation/experiment that could explain the benefit of having this capsule.

We had shown that the capsule influences phagocytosis and replication in *A. castellanii*.

To answer the additional questions asked, we have undertaken several new experiments:

It would be interesting to design Llo WT and ΔctrC expressing GFP (or immunostaining) to decipher their replicative behavior:

are they both in LCV?

- we used a U2OS cell line constitutively expressing Sec61β-GFP for infection assays and to visualize the vacuole. As now shown in the new Supplementary Figure 11, both, the WT and Δ*ctrC* bacteria replicate in a *Legionella*-containing vacuole (LCV) and both recruit ER vesicles to the vacuole as seen by a GFP ring surrounding the bacteria at early timepoints of infection (magnified inlet).

This new experiment is now shown in the new Supplementary Figure 11 also discussed in the manuscript and the discussion.

Lines 301-307 it reads now… To confirm that both *Llo* WT and Δ*ctrC* replicate inside the LCV, we infected U2OS cells constitutively expressing Sec61β-GFP, a marker of the ER. We followed the replication of the WT, Δ*ctrC* expressing dsRed and as

negative control Δ*dotB*, a strain deficient in a functional T4SS, over time (Figure S11). Both, WT and Δ*ctrC* replicate inside the cells building large vacuoles. More importantly, both strains recruit Sec61β early in infection, like *L. pneumophila* for which the recruitment of ER vesicles is a hallmark of intracellular replication.

Lines 476-479 it reads now (discussion)… Furthermore, we show that both WT and capsule mutant recruit Sec61β early in infection (Figure S11). Sec61β is a marker of the ER and recruitment of ER-derived material to the LCV is a hallmark of *Legionella* infection and LCV formation (77). Thus, both the WT and the capsule mutant replicate in the LCV.

Does the capsule favor the survival? Are the bacteria egressing the cells by killing them?

To answer these questions, we measured apoptosis and ROS in living cells during infection using the Opera confocal microscope as well as the replication of the WT and the mutant strain in human primary macrophages (hMDMs).

We tested for the induction of ROS and signs of early cell death by staining hMDMs with CellROX dye and Annexin V in living, infected cells and used the Opera confocal microscope for image acquisition and analyzed 100s of cells. However, we could not detect significant differences in either ROS induction or apoptosis (shown in new Figure S12). Thus, these new data suggest that the capsule provides also a replicative advantage in primary human cells, but without significantly affecting cell death or ROS production of host cells.

These new data are added in the new Figure S12 and as text in the results section and the discussion.

Lines 311-317 it reads…To address the question whether the capsule plays a role in modulating cell death, we stained living, infected hMDMs for annexin V, an early marker of apoptosis. However, we did not observe significant difference in annexin V labelling of WT or Δ*ctrC* infected cells (Figure S12A). In addition, we tested whether the capsule may influence the induction of reactive oxygen species (ROS) in hMDMs. We labelled hMDMs with CellROX live cell dye to follow ROS production in infected cells, but we did not observe a significant difference in ROS production (Figure S12B).

Lines 480-484 (discussion) it reads now… However, we did not observe any differences in apoptosis or ROS induction between the two strains. This finding may be explained by the fact that both strains harbor a functional T4SS that allows the bacteria to secrete effector proteins into the host cell and to modulate host cellular pathways, such as apoptosis or ROS formation.

To further answer the reviewer’s question, we tested pore-forming activity in our WT and capsule mutant strains as it is known that *Legionella pneumophila* has pore-forming activity to exit host cells, so yes, host cells will be killed. found that both strains induce pore formation at similar levels in C57BL/6 bone-marrow derived macrophages, albeit it is later than a flagellin-deficient *L. pneumophila* strain (see figure below for the reviewer).

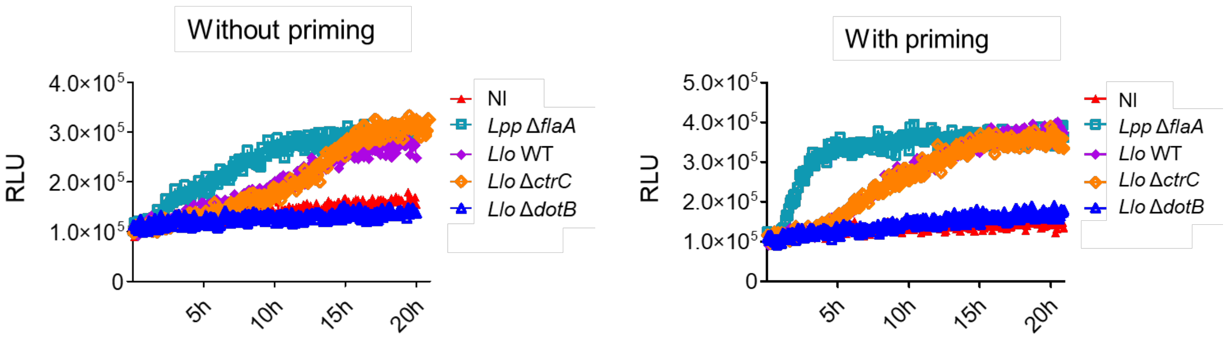

Figure I: *L. longbeachae* induces pore formation in murine bone-marrow derived macrophages. BMDMs from C57BL/6 mice were infected with *L. longbeachae* WT, Δ*ctrC*, Δ*dotB* or with a flagellin-deficient *L. pneumophila* strain as positive control. Cells were either untreated (left panel) or pre-treated with LPS (right panel). Pore formation was measured fluorometrically by uptake of propidium iodide as described previously[1].

In addition, we have analyzed the replication of the WT and the mutant bacteria in human monocyte-derived macrophages (hMDMs). As now shown in new Figure 4J WT bacteria replicate better than Δ*ctrC* bacteria judged by calculating the vacuole size.

These results are added in the new Figure 4J and in the manuscript

Lines 308-311 it reads now… Since *L. longbeachae* can cause the severe pneumonia Legionnaires' disease in humans, we also tested bacterial replication in human monocyte-derived macrophages (hMDMs). We observed that the WT replicates more efficiently in hMDMs than the capsule mutant as indicated by a larger, bacteria containing vacuole size in the WT infected cells (Figure 4J).

Lines 484-488 (discussion) it reads now… Our data also point towards a difference in replication phenotypes depending on the infection models used. We observed a replication defect for the capsule mutant in primary cells such as hMDMs and amoeba as well as *in vivo*, but not in cell lines *in vitro* (Figure S12). This result might point to the known metabolic differences between primary cells and cell lines, a topic that warrants further research.

The M&M section was updated accordingly.
Lines 713-722 it reads now…

Infection of U2OS cells

Human osteosarcoma epithelial cells stably expressing Sec61β-GFP (U2OS-Sec61β-GFP) for labelling of the Endoplasmic Reticulum (107) were cultured in DMEM + GlutaMAX (Life Technologies), supplemented with 10% heat-inactivated FBS (Life Technologies). 20000 cells were seeded in DMEM in 96-well Greiner µClear plates and allowed to adhere before infection. Cells were infected with *L. longbeachae* at MOI 100 for two hours and extracellular bacteria were washed off using PBS. Cells were stained with Hoechst dye (Invitrogen, H3570) at 1 µg/ml to label nuclei. Live imaging was performed at 63x magnification at 37°C and 5% CO_2 using the Opera

[1] Mascarenhas DPA, Cerqueira DM, Pereira MSF, Castanheira FVS, Fernandes TD, Manin GZ, Cunha LD, Zamboni DS. Inhibition of caspase-1 or gasdermin-D enable caspase-8 activation in the Naip5/NLRC4/ASC inflammasome. PLoS Pathog. 2017 Aug 3;13(8):e1006502.

Phenix confocal microscope (PerkinElmer). Images were analyzed using the Harmony® high-content analysis software (PerkinElmer).

Lines 775-787 it reads now
Bacterial replication, early apoptosis, and ROS production in hMDMs
Human monocyte-derived macrophages were seeded in XVivo medium (Lonza) at 37°C and 5% CO_2. Cells were stained with CellTracker Blue (CMF2HC, Invitrogen) and Hoechst dye (Invitrogen, H3570) for 1 hour pre-infection, before washing the cells in PBS. Cells were subsequently infected with *L. longbeachae* expressing dsRed (plasmid SW001, kind gift from Hubert Hilbi) at MOI 10 for 1 hour and extracellular bacteria were removed by washing with PBS. To monitor ROS production, cells were stained with CellROX DeepRed (C10422, Invitrogen) for 30 minutes and dye was washed off as indicated by the manufacturer. To monitor early apoptosis, cells were stained with Annexin V AlexaFluor 488 dye for living cells (A13201, Invitrogen). Live cell imaging was performed using the Opera Phenix confocal microscope (PerkinElmer) and bacterial replication, early apoptosis, and ROS production in single infected cells were analyzed using the Harmony® high-content analysis software (PerkinElmer).

Is the capsule used for abiotic adherence?

We had already before tried to test biofilm formation. However, to obtain single bacteria biofilms from *Legionella* is very difficult. Indeed, despite several attempts by the first and co-authors of this study, we have not succeeded in growing a *L. longbeachae* biofilm in plates. Thus, we cannot experimentally answer the question whether or not the capsule influences abiotic adherence.

Reviewer #2: There are couple of 'straightforward' experiments that would significantly strengthen the MS, as they would interconnect some of the observations made in this MS and provide more insights into the virulence mechanism of the Llo capsule. While desirable, I don't consider these experiments essential for acceptance of the MS:

- data in Fig6 indicate that the Llo capsule reduces immune stimulation as assessed by IL-6 and IL-1beta secretion in murine DCs and BMDMs. Do these immunomodulatory activities observed in cell culture correlate with similar observations in vivo, possibly indicative of a causative link? I think the study would be significantly strengthened if cytokines were measured in BALF of Wt vs ctrC mutant infected animals

As requested by the reviewer, we have now also tested cytokine induction in the BALFs of infected mice and in addition lung damage and protein levels. We observed differences in IL-6 but not TNF, and higher lung damage and protein levels.

These data are added to figure

Lines 385-390 it reads now…Further, we tested cytokine levels in BALF fluid from *Llo* WT or Δ*ctrC* infected mice. Here we observe higher IL-6 induction in the WT as compared to the mutant, but no difference sin TNF-α levels in BALFs of infected mice

at 72 hours post-infection (Figure S15C, D). However, we detected higher protein levels in BALFs of WT infected mice as measured by Bradford assay (Figure S15E). These data indicate that proinflammatory cytokine induction *in vivo* is due to higher lung damage caused by the WT as shown by protein levels and lactate measurements.

Lines 521-525 (discussions section) it reads now… Additionally, we observed that the complemented capsule mutant induces lower TNF-α than the capsule mutant harboring the empty plasmid. *In vivo* we see a reversed phenotype, the WT induces higher proinflammatory cytokine than the capsule mutant (Figure S15A-D). This is likely linked to the WT causing a high degree of lung damage (Figure 4E and S15E).

- it is intriguing that the ctrC mutant has a cell-autonomous growth defect in amoeba but not in the tested mammalian cells. Why would that be? And by what mechanism would the capsule provide Llo with a competitive advantage within amoeba (but not mammalian cells). It would be helpful if the authors could provide some discussion on this topic (and maybe streamline the current – somewhat meandering – version of the MS Discussion

As requested, we have streamlined the discussion and changed the order of certain paragraphs. Furthermore, as described above, we have now tested the growth of the WT and Δ*ctrC* in human, primary cells (hMDMS). In these cells, the *ctrC* mutant shows a growth defect Figure 4J, like in amoeba. Thus, the lack of growth defect in THP-1 cells might be due to the fact that it is a cell line, and that it has e.g. an altered metabolism. It is known that the growth in primary cells is not necessary reflected in cell lines. This has been added to the discussion.

Lines 484-488 it reads now… Our data also point towards a difference in replication phenotypes depending on the infection models used. We observed a replication defect for the capsule mutant in primary cells such as hMDMs and amoeba as well as *in vivo*, but not in cell lines *in vitro* (Figure S12). This result might point to the known metabolic differences between primary cells and cell lines, a topic that warrants further research.

- Is the cytokine phenotype functionally linked to the block in phagocytosis? A simple cytoD experiment would address this question

We refrained from blocking phagocytosis and measuring cytokines as blocking actin polymerization with cytochalasin D likely also influences the turnover of pattern recognition receptors such as TLRs, which artificially skews the cytokine expression in cytoD-treated cells.

Reviewer #3: Major comments

1. Fig 4E, F, how was statistical analysis and standard deviation done on n=2? Surely n=3 is the minimum for this calculation.

The initial calculation was done on the slope of the growth curves, not on the sample number itself. To address the reviewer's comment (and reviewer #2's comment), we

have now performed a new competition assay with n=3 replicates, and more time points sampled. This shows that the WT outcompetes the capsule mutant within 24-48 hours. This result is now presented in the new Figure 4H (shown as competitive index as usually done).

2. Fig. 4D, does the capsule mutant survive and persist in broth similar to wt over this time, ie. is the effect just due to the presence of amoebae?

We are not sure that we understand this question correctly. If the reviewer was inquiring about growth in broth culture, we kindly refer the reviewer to Supplementary Figure 1 where we had shown that there is no difference in growth. If the reviewer was inquiring about potential growth in the infection buffer used for amoeba infection, then the answer is that the bacteria can persist in the infection buffer, but they cannot replicate in this medium, they need to infect amoeba to replicate.

3. Although the authors show that phagocytosis of delta CtrC is increased in THP-1 cells, the mutant shows no reduction in replication in these cells and BMDM. The mouse virulence effects would suggest otherwise. I'm not sure I understand the explanation for this in the sentence (line 241) "In contrast, when we infected human macrophage-like THP-1 cells or murine bone marrow-derived macrophages in vitro, we observed only a slight replication defect of the capsule mutant as compared to the WT, indicating that successful infection by L. longbeachae depends on the interaction of immune cells with the capsule in vivo" What is the evidence for this, what immune cells are implicated here, or is the capsule mutant simply more susceptible to complement mediated bacterial killing in vivo? This could at least be tested in vitro

To answer the reviewer's comment, we have tested complement-mediated killing *in vitro* using bacteria grown to post-exponential growth phase (shown below). We have tested different concentrations of serum ranging at 20%, 50% and 90% serum for 1 hour, 3 hours and 6 hours of treatment. As we did not see any difference between the strains, we also tried adding sucrose to help rupture eventually disintegrated cells (as shown below for the reviewer).

However, all the set-ups we have tried did not show any difference in complement-mediated killing between the strains. This may be explained by the fact that the mutant still contains an intact O-antigen chain (as shown in Figure 2C-D of the MS), that may aid in complement resistance. Unfortunately, we never succeeded in generating mutants in the LPS synthesis genes despite numerous attempts in the lab, as it is very complicated to obtain mutants in *L. longbeachae* (to our knowledge only two mutants exist: a *dotB* mutant constructed in the lab of H. Newton and our *ctrC* mutant).

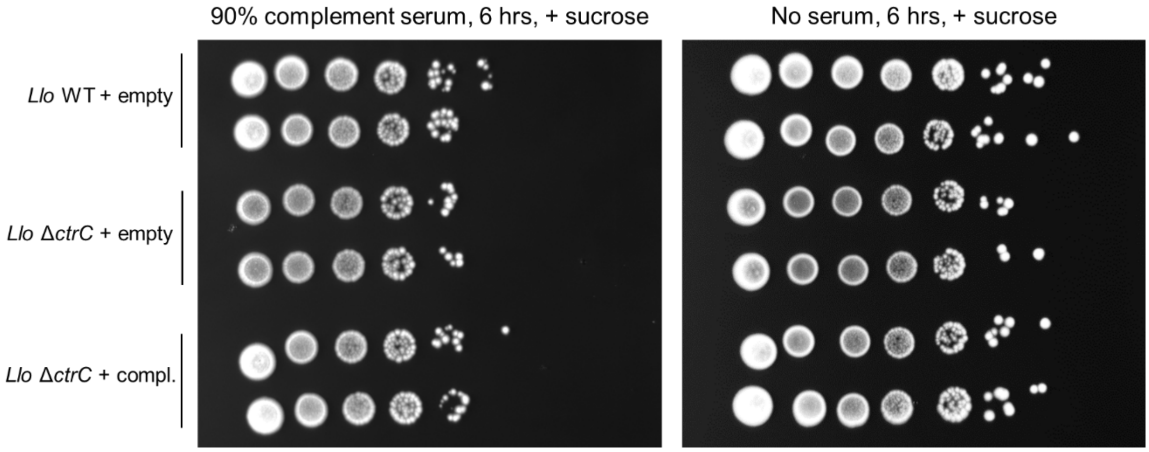

Figure II: The *L. longbeachae* cell wall protects from complement-mediated killing independently of the capsule. Bacteria were grown to PE phase in BYE liquid broth, washed in PBS (containing $MgCl_2$ and $CaCl_2$), and treated with or without 90% human complement serum (Sigma S1764) in PBS for 6 hours at 37°C. Subsequently, additional 0.25 M sucrose were added for 30 minutes and bacteria were plated in serial dilutions on BCYE plates and grown for 4 days at 37°C.

4. In Fig 5 and 6, the authors move away from THP-1 cells to primary macrophages and murine bone-marrow derived dendritic cells. These experiments have not controlled for bacterial load. If the increased phagocytosis in THP-1 cells of the CtrC mutant is consistent in these cells, this would directly relate to the cytokine response. ie. increased bacterial load leads to increased production of cytokines. Hence the idea that the capsule inhibits cytokine production or contributes to a low cytokine level is not correct.

In the manuscript, only Figure 6 showed data on primary human or murine cells. We controlled for bacterial load by meticulously adjusting for OD at several dilution steps when preparing the bacterial dilutions. In addition, we included six different donors to account for potential deviations in infectious loads.

5. Related to the point above, have the authors tested cell death of macrophages upon infection with wt and the ctrC mutant, which could also explain differences in cytokine secretion

As requested by the reviewer, we have now tested cell death in live hMDMs infected with the mutant and the WT by staining with annexin V, a known indicator of early cell death. However, we did not detect any significant differences between the WT and the capsule mutant.

These new data have been added in the new Figure S12.

In addition, we tested pore-forming activity in the WT and capsule mutant strains and found that both strains induce pore formation at similar levels in C57BL/6 bone-marrow derived macrophages, albeit later than a flagellin-deficient *L. pneumophila ΔflaA* strain (see below). In addition, this pore-forming activity is dependent on the T4SS, as the *ΔdotB* strain fails to induce pore formation.

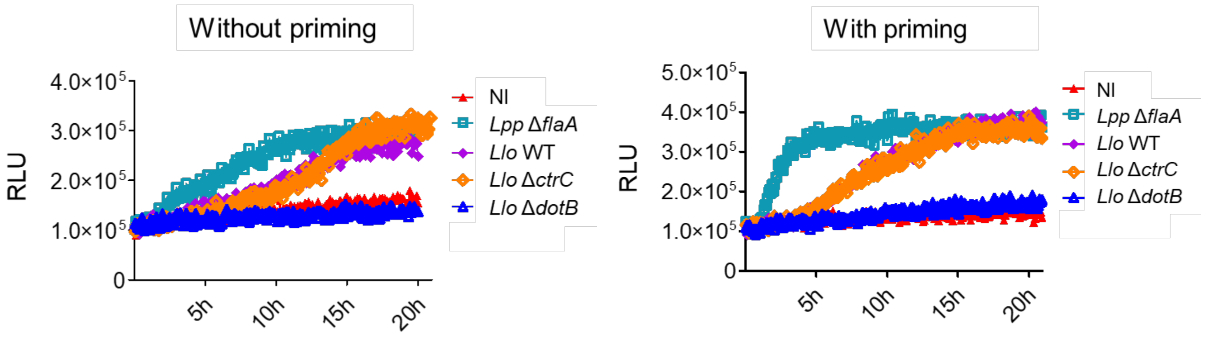

Figure I: *L. longbeachae* induces pore formation in murine bone-marrow derived macrophages. BMDMs from C57BL/6 mice were infected with *L. longbeachae* WT, *ΔctrC*, *ΔdotB* or with a flagellin-deficient *L. pneumophila* strain as positive control. Cells were either untreated (left panel) or pre-treated with LPS (right panel). Pore formation was measured fluorometrically by uptake of propidium iodide as described previously[2].

6. Fig. A-C, why was fold change used here and not pg/mL which is the usual way to express a cytokine response. Biological relevance is much easier to judge with the latter.

We decided to show data as fold change, as we observed, as expected, differences between the six donors used for this experiment. However, the trend between individual donors is similar in that the capsule mutant induces lower IL-6 levels than the WT or the complemented mutant. Given that we used primary hMDMs, these deviations can be explained in that some donors react to infection with high cytokine production as compared to others that showed a lower cytokine response.

7. Line 333, I'm not sure I understand statement that "IL-6 and TNF are highly induced upon infection with the L. longbeachae ΔdotB mutant. However, TNF and IL-1b secretion in human macrophages does not seem to be driven by the capsule but are rather dependent on the presence of a functional Dot/Icm T4SS" when there is no difference statistically and huge variability in data points among the strains/samples (Fig6A-C). The only significant result shown was in IL-6 between the capsule mutant and complemented strain (not even between the wt and crtC mutant). I think these results have been overinterpreted.

The cytokine data from hMDMs have been normalized to the WT as the reference. Thus, since the WT data is not normally distributed, we performed statistical analysis between the empty mutant and the complemented mutant. Thus, we do not see overinterpretation in our data.

[2] Mascarenhas DPA, Cerqueira DM, Pereira MSF, Castanheira FVS, Fernandes TD, Manin GZ, Cunha LD, Zamboni DS. Inhibition of caspase-1 or gasdermin-D enable caspase-8 activation in the Naip5/NLRC4/ASC inflammasome. PLoS Pathog. 2017 Aug 3;13(8):e1006502.

Part III – Minor Issues: Editorial and Data Presentation Modifications
Please use this section for editorial suggestions as well as relatively minor modifications of existing data that would enhance clarity.

Reviewer #1: The capsule is known to protect bacteria from antibiotics. Have you tried to test the potential sensitivity of the capsulated/non-capsulated strains for several antibiotics?

As requested by the reviewer we have tested several antibiotics on bacteria expressing the empty vector or complementation vector (shown below for the reviewer). We included polymyxin B, a known disruptor of Gram-negative cell walls (disrupts LPS integrity). Of note, it is known that *Legionella pneumophila* is resistant to polymyxin B[3]. Our data show that also *L. longbeachae* is resistant to polymyxin B, which can be explained by the fact that we did not manage to disrupt the O-antigen production in *L. longbeachae* and Figure 2 of the manuscript shows that the O-antigen between the WT and Δ*ctrC* looks structurally similar. As shown for the reviewer in the figure below, there are no differences in antibiotics resistance to the tested antibiotics between the WT and the capsule mutant.

[3] Robey M, O'Connell W, Cianciotto NP. Identification of Legionella pneumophila rcp, a pagP-like gene that confers resistance to cationic antimicrobial peptides and promotes intracellular infection. Infect Immun. 2001 Jul;69(7):4276-86. doi: 10.1128/IAI.69.7.4276-4286.2001.

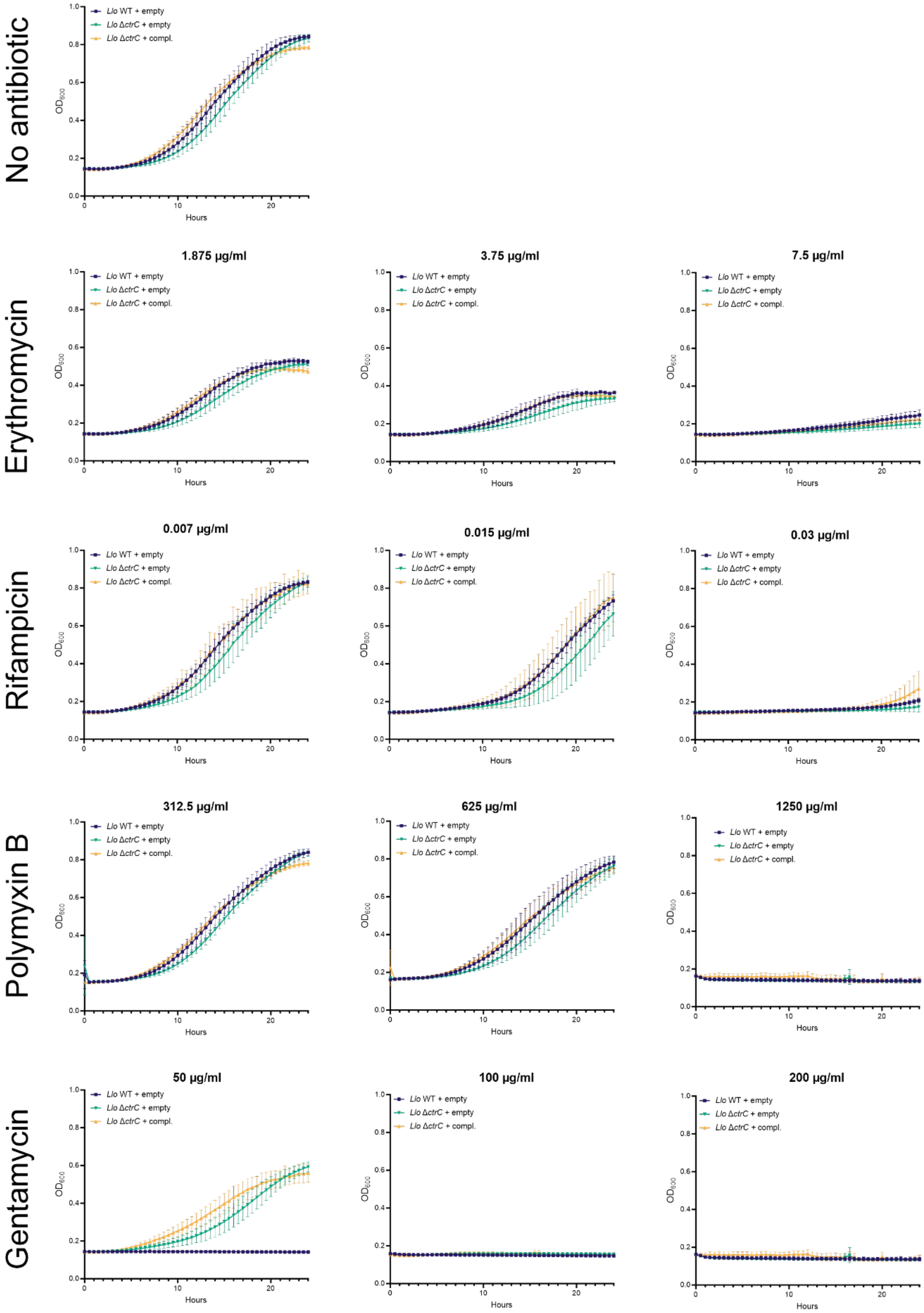

Figure III: The capsule does not seem to influence susceptibility to select antibiotics. Bacteria were grown in 96-well plates in BYE-chloramphenicol medium or with additional antibiotics at the indicated concentrations. Growth was monitored by measuring OD_{600} in a Tecan Infinite M Nano plate reader.

The paragraph “Encapsulated L. longbeachae is more sensitive to salt stress” could be placed after the “The highly anionic L. longbeachae capsule is resistant to silver staining.” as it interferes with the reading of the in vivo/in cellulo section.

We thank the reviewer for this suggestion. The paragraph has been moved to follow the paragraph on silver staining (lines 201-223). The order of supplementary figures has been changed accordingly, Figures S9 and S10 are now S5 and S6, respectively. All the following supplementary figure numbers have been changed accordingly.

Line 39-40/53: The word “crucial” is overstated.

This was changed as requested.
Lines 39+53. It reads now “important” instead of “crucial”

Line 49: Missing “.”

Changed

Line 64: Rephrase – Gardening activity is a risk factor.

This was rephrased as requested.
Lines 64-65 it reads now…Thus, gardening can constitute a risk factor for infection with *L. longbeachae*

Line 85: Ref after infection

Now line 86 Reference 21 (Massis *et al.*) inserted

Line 106: Wild type is written in line 84 and an abbreviation should be put here.

Now line 85 and 107 it was corrected accordingly

Line 130-132: Rephrase, the word “indicate” should be replaced by “suggest”.

Changed as requested.

Lines 131-133 it reads… This extremely high conservation in gene content, sequence similarity, and in the genomic location suggest that the acquisition of the capsule cluster dated back to a common ancestor of *L. longbeachae*.

Line 140-142: The conclusion seems too strong compared to the data, you could qualify it.

Line 142 it reads now… “Thus, the capsule cluster may have been acquired from soil-dwelling bacteria.”

Line 166-168: “80% of the cells express the capsule…” instead.

Changed as requested. Lines 166-168 now reads… However, 50% of the complemented capsule mutant strains expressed a capsular structure indicating that regulation of capsule expression may be more complex (Figure S3C).

Line 168-170: Rephrase the conclusion.

This was rephrased as requested.
Lines 169-171 it reads now… Taken together, our results confirm that, in contrast to *L. pneumophila*, *L. longbeachae* expresses a capsule and that *ctrC* is mainly responsible for its expression on the cell surface.

Line 230: “and” instead of “at”

Now line 260, corrected

Line 652: Typo

Now line 724, corrected

Line 1091: “images”

Now line 1178, corrected

Line 1116: ΔctrC

Now line 1216, corrected

Line 1119: “MOI XYZ”

Sorry for this oversight this was corrected.
Now line 1215, it reads “MOI 0.1”

Line 1134: ΔctrC or ctrC mutant

Now line 1232, corrected to “Δ*ctrC*”

Correct “gentamycin/gentamicin” in the text and figure legends.

Corrected in all instances

References should be fixed (ex ref 36, 41, 78)

The references were corrected

Figure 1: The names of the genes are missing.

We apologize for the missing gene names. Figure 1 was replaced by a corrected version including the gene names. The Figure legend was changed accordingly.

Lines 1166-1169 it reads now… For reference, the *L. longbeachae* NSW150 capsule cluster genes and gene names are shown on the top of the figure. Color code: blue, pathway genes for nucleotide sugar precursor synthesis; yellow, putative transportation genes; green, putative glycosyltransferases; grey, genes of unknown function.

Figure 2: C) “100” on the ladder

We thank the reviewer for bringing this our attention. We replaced the figure without “100” on the ladder.

Figure S2: ΔctrC

Now line 1265, corrected to “Δ*ctrC*“

Figure S3: The legend doesn’t match the figure.

Thank you for catching this, we had inadvertently swapped subsections B) and C) This was corrected.

Figure S4: “Llo WT; red line” but it appears black on the figure.

We apologize for this oversight. We have changed to “green” and indicated it in line 1286.

Figure S8: Y-axis for the two figures is different. The y-axis of FigS7B is not expressed in log10CFU, is it on purpose?

Now Figure S13: Both axes are now labelled the same way. It reads “$\log_{10}$ CFU”.

Figure 2A/B: Have you tried to knock-out ctrB, ctrD or bexD to see if the capsule was missing and then do a complementation? The complementation only restores 50% of the phenotype, if you need two other genes to restore the capsule expression, your ctrC deletion might have impeded the expression of the genes downstream as well. These points should be discussed later in the manuscript as several other genes could be involved in the phenotype observed. “ctrC is mainly responsible for its expression on the cell surface” is then an overstatement at this stage.

We have attempted multiple times to generate other mutants in the genes mentioned above. However, these attempts were not successful neither within the CPS nor within the LPS/O-antigen gene clusters. As explained above, making mutants in *L. longbeachae* is very difficult. It took a year to get the *ctrC* mutant and to our knowledge only two mutants in *L. longbeachae* exist: ours and the *dotB* mutant constructed by H. Newton’s group.

We do not think that this is an overstatement as the *ctrC* mutant clearly has no capsule as shown in the electron micrographs (Figure 2A) and complementation can restore capsule expression on the surface (Figure 2B).

Figure 2C/D: The capsule characterization should be strengthened. Are the exopolysaccharides surface-associated and/or secreted? Could the PS observed on WesternBlot? What are the genes involved in the first step of the synthesis of the polysaccharides?

Our EM data show that the capsule is surface associated. Unfortunately, we cannot perform Western Blot on the CPS as there is no antibody available. If we write as requested by the reviewer about the first step of CPS biosynthesis this would all be speculation, as further experiments would be possible, but due to the lack of other mutants in biosynthesis genes (despite several attempts at generating these mutants as explained above) could not be done. Thus, we prefer not to speculate.

Figure S2: Please add the strain ΔctrC+empty as it is used later in the manuscript.

The empty and complemented strains have been added to Figure S3, now as panel S3D.

Figure 3A/Figure S4: A control of the RNAseq could be included with RTqPCR on a few genes (for example ctrC, ctrD and bexD). As stated in the M&M, the exponential phase is reached at OD600 2.0-2.5 and the post-exponential phase OD600 3.7-4.2 but the stationary phase of the stains is obtained OD600 around 1.2 in FigS2. Is OD600 2.0-2.5 truly an exponential phase?

Yes, OD600 2.0-2.5 is truly exponential phase. The experiments shown in figures 3A+S7 versus S2 have been carried out using bacteria grown either in Erlenmeyer flasks or 96-well plates, respectively. The growth in Erlenmeyer flasks supports growth to higher OD due to better aeration of the bacterial cultures as compared to 96-well plates, which is why we used Erlenmeyer flasks for our RNAseq samples, and the OD at PE phase is higher than in the plate reader.

We are surprised by the request of the reviewer to confirm RNAseq data by qRT-PCR as this was necessary for microarray analyses but is not anymore for RNAseq data. However, to address the reviewer’s comment, we have performed qRT-PCR on genes of the capsule transporter operon (*bexD-llo3154*). We compared mRNA levels of WT or Δ*ctrC* bacteria grown to either exponential (E) or post-exponential (PE) growth phase. Data were normalized to *tldD* as the house-keeping gene and are shown below as relative mRNA levels compared to the WT E phase levels. Our data qRT-PCR data confirm that the capsule transporter operon is highly transcribed in E phase and downregulated in PE phase both in WT bacteria and the capsule mutant (see below figure for the reviewer). We detect a low mRNA signal in the *ctrC* mutant for *bexD*, which may be due to an internal transcriptional start site in the upstream *ctrD* gene. Data are shown as means ± SD of n=3 experiments. (The results below are shown for the reviewer).

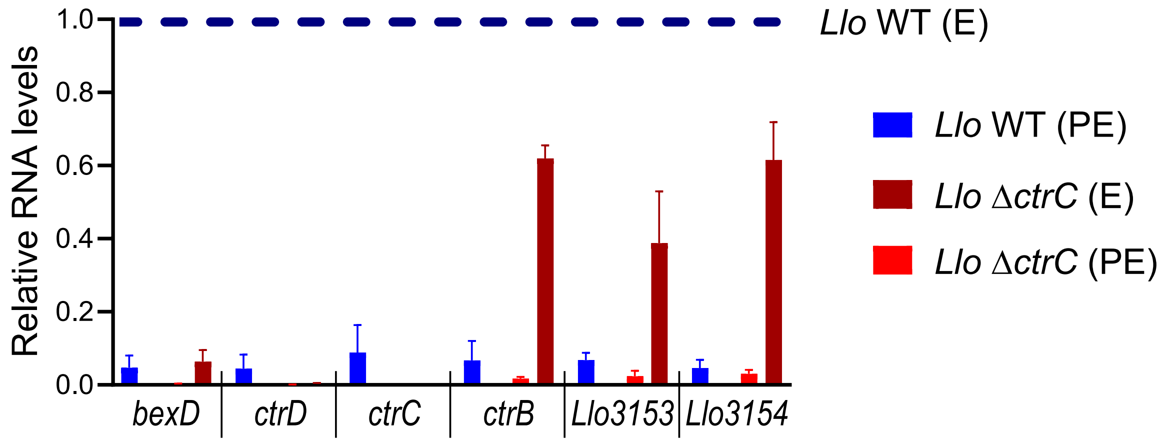

Figure IV: qRT-PCR of *L. longbeachae* capsule transporter operon. Total RNA was isolated from *L. longbeachae* WT and Δ*ctrC* strains during exponential (E), post-exponential (PE) phase. qRT-PCR data are represented as fold changes of gene expression between exponential and post-exponential phase, relative to *L. longbeachae* WT exponential phase. *tldD* was used as the housekeeping gene and data shown correspond to means ± SD of n=3 experiments.

Figure S5: The mutant being ΔctrC, I would expect no data for the gene.

Now Figure S7. There seems to be a misreading of the figure. The reads are compared to the WT as the reference. Thus, what we see in this figure effectively is gene expression of *llo3151* of the WT.

Line 204-206: The statement "This showed that almost all genes of the capsule cluster in the L. longbeachae WT strain were significantly downregulated in PE as compared to E phase (Figure 3A). This correlates with the visualization of the capsule in the PE phase (Figure S3A)." is challenging to apprehend. Is the expression of these genes that delayed compared to their transcription (timewise)? Overall, it would have been interesting to have an RNAseq on the intracellular bacteria at different time points to strengthen the statement.

We apologize for the confusing wording. We have changed the sentence to make it clearer.

Lines 232-237 it reads now… This showed that almost all genes of the capsule cluster in the *L. longbeachae* WT strain were significantly downregulated in PE as compared to E phase (Figure 3A), indicating that the expression of the capsule on the cell surface is growth phase dependent. However, the capsule is visible only in the PE phase (Figure S3A) suggesting that the building of its complex polysaccharides and their export to the cell surface takes time and thus expression of the CPS (Figure 2) is highest in later growth phases.

Figure 3B: The authors should provide a quantification of the mKate2/GFP of the bacteria in several infected cells.

As requested by the reviewer, we had already tried to quantify mKate/GFP, however, unexpectedly, the degradation tag, that works perfectly in *L. pneumophila*, did not work

in *L. longbeachae* (see figure below, the GFP signal shows a clear rise and decline during growth of *Lpp* WT but not for *Llo*). However, similar to our findings from infected cells, the GFP signal steadily increases in the *Llo* WT and as our control plasmid is not producing GFP in infected cells (as shown in the MS), we conclude that the capsule is transcribed intracellularly but we cannot resolve the timely regulation of its expression in infection. To make it clearer we deleted the mention for the degradation tag as it did not work from the manuscript and explained better what we did.

Lines 252 – 255 it reads now… We detected GFP expression starting at 12h that continuously increased in intracellularly replicating bacteria containing the cap_{prom} promoter construct, but not the negative control. This revealed that the capsule is transcribed in both eukaryotic hosts. Thus, the capsule is universally transcribed *in vitro* and upon infection.

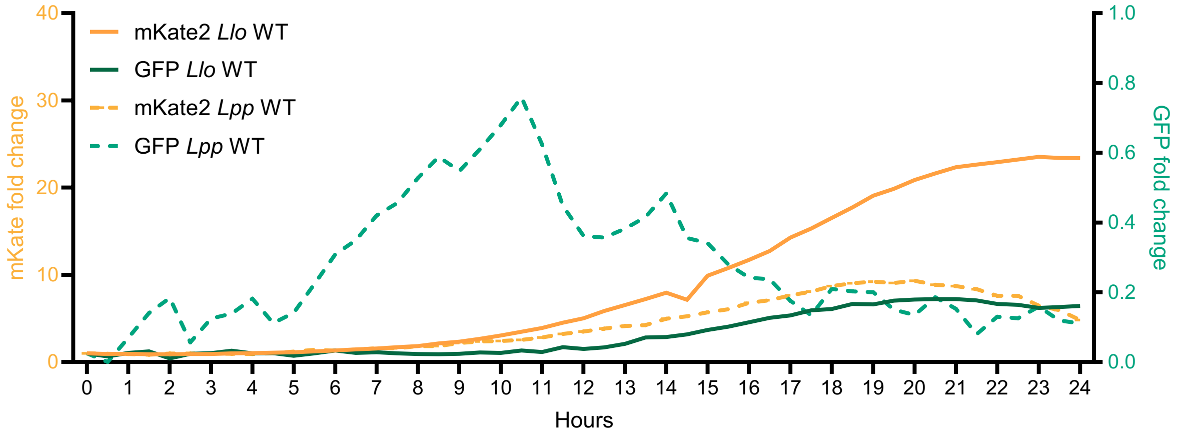

Figure V: Dual reporter expression during growth in BYE medium. *L. longbeachae* WT (straight lines) and *L. pneumophila* WT (dashed lines) expressing the dual reporter plasmid were grown in BYE medium and fluorescence of mKate2 and GFP was measured using a Tecan Infinite M Nano plate reader. Data shown means of n=2 experiments, normalized to t=0.

- Some bacteria seem to express mKate2 but not sfGFP, are those extracellular or intracellular?

These bacteria are extracellular, but more importantly they are out of the focus plane as indicated by the "tail" of mKate2. Thus, it cannot be stated with certainty that these bacteria do or do not express sfGFP. However, we also see GFP-expressing bacteria outside of the cells, likely due to GFP not being degraded fast enough in *L. longbeachae* (see Figure V above).

- Does an antibiotic was used to kill extracellular bacteria for this experiment (not written in the M&M)? If gentamicin is used to kill extracellular bacteria; the MIC of each strain should be assessed.

Bacteria were washed off by extensive washes in PBS and no antibiotic was used, thus there is no antibiotics indicated.

- Is the GFP detected at 14 hours for the THP-1 and the amoebae? Does the GFP signal change over time? The authors are showing 22hrs and 24hrs images in

Figures 3B and S6 but the GFP signal is stated to be detected at 14hrs. I would recommend showing a figure of the timelapse instead of just one time point.

To answer this question, we have made a film of the infection of THP-1 cells over 2-23 hours. One can see that GFP expression starts at 12h. Four new videos are now added, pointed to in line 251.

The new video is added to the supplementary file

- This experiment is carried out with PE-phase bacteria which, as indicated in the manuscript, express the capsule, have you tried using E-phase bacteria?

We used PE phase bacteria to allow for capsule expression on the cell surface. In general, E phase bacteria are not infecting well, which is why we grow them to PE phase as well.

Figure S6: The shape of the amoebae is concerning and raises the question of their viability. Most of the bacteria seem to be extracellular, this experiment should show intracellular bacteria and a quantification.

We apologize for not having shown nicer images. We have changed the images now and have enlarged them. The amoeba rounds up when they are infected and due to the infection buffer, which is “stress condition” for amoeba.

New Figure S8 shows these images.

We have tried to quantify intracellular bacteria in *A. castellanii* using the Opera confocal microscope and by overlaying the amoeba with low melting agar to fix them in the focal plane. However, even when using agar overlay, the cells migrate out of the focal plane, and we cannot quantify bacterial expression of mKate2/GFP in the same cell over time.

- Can you explain why the experiment is done at 37°C while amoebae are usually kept at 20-25°C?

It was done at 37°C to speed up the expression of the sfGFP reporter. However, as suggested by the reviewer, we repeated the infection at 25°C and found that GFP was also expressed upon infection of *A. castellanii* at 25°C.

New Figure S8 shows these images, and it is discussed in the manuscript.

Lines 193-196 it reads now… To test whether capsule expression may be temperature-dependent, we grew *Llo* WT, Δ*ctrC* and complemented Δ*ctrC* at 20°C in liquid medium. CPS extracted from bacteria grown at 20°C showed that also at 20°C the capsule is expressed in PE phase only in the *Llo* WT and complemented Δ*ctrC* (Figure S4C).

- Could the temperature influence the synthesis of the capsule?

To answer this question, we have now tested the expression of the capsule also at a growth temperature of 20°C by SDS gel electrophoresis and Stains-all staining. We observed that the capsule is also expressed at 20°C in bacteria grown in liquid medium

to PE phase, shown in new Figure S4C) (and at 37°C, as shown in the main Figure 2 in the MS). Thus, we conclude that between 20°C and 37°C, the capsule is equally expressed on the bacteria in post-exponential growth phase.

We have added these new data in the new Figure S4C.

Figure 4 C/D: The experiment in Fig4C shows ΔctrC replication compared to the ΔctrC+empty in FigD. These experiments should be done with both ΔctrC and ΔctrC+empty strains to exclude any effect of the empty plasmid.

Now Figure 4F+G. The experiment in figure 4F shows strains without any plasmids (both Δ*ctrC* and Δ*dotB* are genomic mutants). Naturally, any plasmid will have a fitness cost on bacterial growth, which is why we compared strains harboring the plasmid (empty or complemented) in figure 4G.

- Why different MOI were used for Fig4C/D experiments (figure legend)? Can you comment on why changing the MOI seems to influence the replication of ΔctrC.

Now Figure 4G. The MOI had to be increased for figure 4G, as the pBCKS plasmid lowers the fitness of the bacteria and one does not detect countable CFUs at lower MOI when selecting on BCYE-chloramphenicol plates.

- Furthermore, the ΔctrC+compl strain is showing the same behavior as Llo WT while it has been shown that only 50% of the capsule is restored and 60% of the mice are surviving, can you comment?

Now Figure 4G. Compared to the mutant strain, *L. longbeachae* WT and Δ*ctrC* + complemented strains are similarly phagocytosed by amoebae; however, this information is not specifically indicated in the graph as data are plotted relative to time point 1h after infection (see Figure 5 for discussion on this topic). A clear difference in bacterial survival between these two strains, however, is evident at 24h post infection, with a stronger decrease in the CFU counts of the complemented strain, compared to the WT. Given that the capsule is partially restored in the complemented strain, this phenomenon is probably due to amoeba elimination of the complemented bacteria that do not efficiently express a capsule, while those with their capsule restored can compete and survive. Then, at later time points, these capsule-expressing bacteria can recover, showing a similar behavior as the WT.

Overall, the experiments on amoebae need to be strengthened, for example with microscopy images and quantification of intracellular replication to match the paragraph's title.

Now Figure 4F-H. We are a little confused by this comment as we show microscopy images. In addition, we provide data on intracellular replication in amoeba in figures 4F, 4G, and 4H.

Figure 4A/S7A: Is there a particular reason why you used 2 different bacterial loads? Do you have CFUs for the bacterial load of 107 with the ΔctrC+compl?

We used two different bacterial loads, to test if a lower infection dose also causes a virulence phenotype in mice. We did not plate the CFU for ΔctrC+compl but normalized meticulously over OD.

Figure 4F: The western blot and the flow cytometry data should be provided in a supplemental figure.

The gating strategy for flow cytometry and Western Blot showing BlaM expression in the different *L. longbeachae* strains are now shown in Supplementary Figure S10A+B.

Figure 5: The authors should include the complemented strain for these experiments.

We prefer to not add the complemented strain to this experiment as we then have to use WT and mutant strains that carry a plasmid, and as plasmids in *Legionella* have a huge fitness cost, the results can be difficult to interpret afterwards. Furthermore, we have shown at several instances that the complementation is only partial, thus we prefer to leave the figure as it is.

Figure S7B: The CFU of t=0 should be provided. I would recommend a later point for the ΔctrC to know if the mice are clearing the infection or if the infection is stabilized over time

The mouse data were obtained by the lab of Dario Zamboni in the standard way he is always publishing. He never provides CFU of t=0 as seen in the below cited papers, one published in PloS Pathogens (DOI: 10.1371/journal.ppat.1007886) and also others: DOI: 10.4049/jimmunol.1501223; DOI: 10.1002/eji.202249985 ; DOI: 10.1093/infdis/jiw560.

The mice infected with the *ΔctrC* strain are clearing the infection but not those infection with the WT strain as seen from the measure of the body weight (figure below for the reviewer).

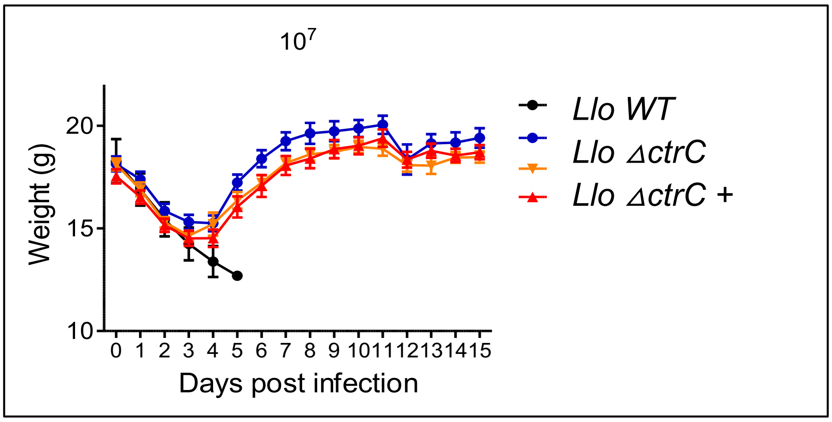

Figure VI: Analyses of the survival of the WT and ***ΔctrC*** **strain in mice over 15 days.** Mice were infected with 10^7 *L. longbeachae* WT (black line) and *L. pneumophila ΔctrC* (blue, orange and red lines) and the body weight of the mice was recoded over 15 days. The WT infected mice succumb to infection after 6 days, in contrast the *ΔctrC* infected mice recover.

Figure S7C: Related to this figure, it would be interesting to know if the capsule of L. longbeachae protects from complement-mediated binding/killing.

As requested by the reviewer, we have tested complement-mediated killing *in vitro* using bacteria grown to PE phase. We have tested different concentrations of serum ranging at 20%, 50% and 90% serum for 1 hour, 3 hours and 6 hours of treatment. As we did not see any difference between the strains, we also added sucrose to help rupture of eventually disintegrated cells. However, in no condition difference in complement-mediated killing between the strains was observed (see Figure below for the reviewer). This may be explained by the fact that the mutant still contains an intact O-antigen chain (as shown in Figure 2C-D of the MS), that may aid in complement resistance. Unfortunately, we did not succeed either in generating mutants in the LPS synthesis genes despite numerous attempts at doing so.

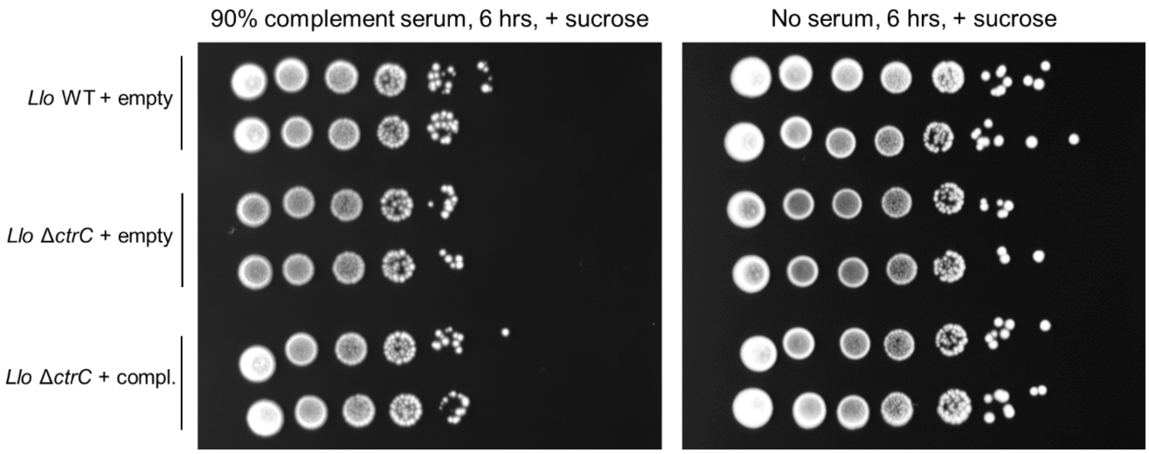

Figure VII: The *L. longbeachae* cell wall protects from complement-mediated killing independently of the capsule. Bacteria were grown to PE phase in BYE liquid broth, washed in PBS (containing $MgCl_2$ and $CaCl_2$), and treated with or without 90% human complement serum (Sigma S1764) in PBS for 6 hours at 37°C. Subsequently, additional 0.25 M sucrose were added for 30 minutes and bacteria were plated in serial dilutions on BCYE plates and grown for 4 days at 37°C.

Figure 6: It would have been nice to know the cytokine production in mice.

As requested, we have tested cytokine induction *in vivo* in BALF from infected mice, using the WT and mutant strain.

Lines 385-390 it reads now… Further, we tested cytokine levels in BALF fluid from *Llo* WT or Δ*ctrC* infected mice. Here we observe higher IL-6 induction in the WT as compared to the mutant, but no difference sin TNF-α levels in BALFs of infected mice at 72 hours post-infection (Figure S15C, D). However, we detected higher protein levels in BALFs of WT infected mice as measured by Bradford assay (Figure S15E). These data indicate that proinflammatory cytokine induction *in vivo* is due to higher lung damage caused by the WT as shown by protein levels and lactate measurements.

Reviewer #2: - Why were only female mice used in this study? Can we expect sex differences?

We used only female mice as housing of male mice is costly and impractical as males need to be housed in solitude and away from female mice. However, it is well known that in humans, males are at higher risk of developing Legionnaires' disease. This may

be an interesting question to address in the future, however, testing sex differences for infection with *L. longbeachae* in mice is beyond the scope of this manuscript.

- legend fig.4 “A. castellanii trophozoites were infected at MOI XYZ” – seems like the placeholder xyz needs to be replaced by actual MOI

We apologize for this oversight. This is now corrected.
Now line 1215, it reads “MOI 0.1”.

- the figures are not always very reader-friendly. The fonts can be too small (especially 3A), the graph lines can be very thin (Fig.4) and the panels not sufficiently self-explanatory (e.g. can the panels in Fig.4 be annotated so it’s immediately obvious whether these experiments were carried out in amoeba or mice without having to refer to the figure legend)

We apologize if the figures were too small. Thus, label fonts and line weights have been adjusted accordingly in Figures 3, 4, S2, S3D, S7, S9, S13. Labels have been added to panels in Figure 4 to distinguish which cell type is depicted.

- along those lines: could the mouse and amoeba data be shown in two separate figures? I would also recommend moving some of the supplementary data into the main figure. FigS7A-C provides more information than current Fig.4A. I would also recommend showing Fig.S8 as a main figure, because I think it is important to contrast the growth defect of ctrC mutanat in amoeba with the lack thereof in macrophages

As requested by the reviewer, we have added Figures FigS7B-C to the main Figure 4, now Figure 4 C, D and have also added new results about lung damage, now Figure 4E.

Lines 269-274 it reads now… *L. longbeachae* WT replicates to higher numbers in the murine lungs as compared to the capsule mutant, but both seem to reach the blood stream as we measured comparable levels of CFUs in the spleen (Figures 4C, D). Furthermore, we measured levels of lactate dehydrogenase (LDH) from bronchoalveolar lavage fluid (BALF) of infected mice to determine whether the capsule plays a role in lung damage. Indeed, the WT induces higher levels of LDH than the Δ*ctrC* strain (Figure 4E), indicating that the capsule is implicated in lung damage.

- Fig.4E y axis label – percent survival of what? Display competitive index data instead?

Sorry for not having been clear. Percent survival refers to the bacterial strains shown in the graph. The figure legend has been changed to clarify that survival of bacteria is shown. Now figure 4H

Lines 1215-1218it reads now… **H)** *A. castellanii* trophozoites were infected at MOI 0.1 with equal amounts of *Llo* WT or Δ*ctrC*. Samples were taken at 1, 8, 24, 30, 48, 96, 120, and 144 hours post-infection and normalized to the input control. Data show the competitive index of *Llo* Δ*ctrC* over *Llo* WT for n=3 independent experiments.

- Fig.4E – at 48 hpi WT completely outcompeted the ctrC mutant; the graph only shows 0 and 48 hpi data (all the later timepoints are superfluous and can be deleted) – to show these data as a line graph, timepoints between 0 and 48 hpi - that actually are intermediate datapoints – need to be included

As requested by the reviewer, a new competition assay has been performed including earlier timepoints between t=0 and t=48 hours post-infection in n=3 independent experiments (shown in new Figure 4H). Our data show, that within 24-48 hours, the capsule mutant is outcompeted by the WT. Furthermore, we now show the competitive index which better depicts the result.

- Fig.4E – this experiment was done as an n of 2?

Yes, this experiment was done twice as the phenotype of the capsule mutant being outcompeted by the WT was so strong. However, to address this comment, the experiment has been repeated with earlier timepoints and n=3 independent experiments (as stated above).

- fig6 figure legend – I think it should be A-C) hMDMS not A- D)

We apologize for the oversight. Now line 1239, changed accordingly.

- Figure 6 – in panels A – C cytokine data is depicted as fold change; in all other panels as mass/ volume (pg/mL). I would recommend showing data as mass/ volume for panels A- C as well, since mas/ volume data are much more informative than fold changes, e.g. by allowing readers to assess whether any of the cytokine measurements are in the physiological range

We decided to show data as fold change, as we observed, as expected, differences between the six donors used for this experiment. However, the trend between individual donors is similar in that the capsule mutant induces lower IL-6 levels than the WT or the complemented mutant. Given that we used primary hMDMs, these deviations can be explained in that some donors react to infection with high cytokine production as compared to others that showed a lower cytokine response.

- ctrC mutant phenotypes were complemented for experiments in in hMDMs (Fig6 A-C) but not in murine DCs (D-F) or murin BMDM (G-I). Ideally complementation should be done for experiments in murine DCs/ BMDMs (D – I) as well

As requested, we tested IL-6 and TNF induction also for the complemented strain and the mutant strain in murine BMDMs. No difference was observed for IL-6 induction, but for TNF.

These results are added in Figure S15A-B and in the manuscript.

Lines 385-388 it reads now… When cytokine induction by the capsule mutant or the complemented mutant was tested in BMDMs, we observed that complementation of the capsule reduces TNF-α levels in BMDMs as compared to the capsule mutant, but it does not influence IL-6 levels *in vitro* (Figure S15A, B).

- Fig. 6 why does Llo not induce TNFalpha in THP1 cells when it does so robustly in murine BMDMs? Could the authors provide a positive control demonstrating that their THP1 cells can be activated for TNFalpha production? Without such a control the data shown in Fig6C are difficult to interpret

Figure 6 only shows cytokine production in primary cells (either human or murine), not THP-1 cells. As we use primary cells and not a cell line for these experiments, we deemed testing LPS activation is not necessary.

Reviewer #3: Minor comments

1. Line 242, there is no difference not a slight reduction in replication (Fig. S8)

This was changed as suggested.
Now line 319 it reads now... “no replication defect”.

2. Line 240 …. indicating that complementation partially restored virulence

This was corrected
Now line 268 it reads… “complementation partially restored virulence”.

3. Line 1119 MOI XYZ?

Sorry for this oversight this was corrected.
Now line 1215, it reads “MOI 0.1”.

4. Line 1121 percentage survival of amoebae or bacteria?

This is explained now.
Now lines 1217-1218 it reads now… Data show the competitive index of *Llo ΔctrC* over *Llo* WT for n=3 independent experiments.

5. Fig. 4F. What does percent secretion mean? This result is usually expressed as ratio blue/green fluorescence I think

Now Figure 4I. As requested this was changed to ”% cells with cleaved CCF4”, as previously published article by Schator *et al*., 2023 (https://doi.org/10.1038/s41467-023-37885-z).

6. Line 1144 …A-C not A-D

We apologize for the oversight. Now line 1242, changed accordingly.
